# Analytical Methods for the Characterisation of Aroma Compounds in Milk

**DOI:** 10.3390/foods15111885

**Published:** 2026-05-27

**Authors:** Kevin Ghavalas, Sonja Kukuljan, Yada Nolvachai, Snehal R. Jadhav, Daniel A. Dias, Russell Keast

**Affiliations:** 1 ARC Training Centre for Hyphenated Analytical Separation Technologies (HyTECH), Burwood, VIC 3125, Australia; sonja.kukuljan@noumi.com.au (S.K.); y.nolvachai@deakin.edu.au (Y.N.); snehal.jadhav@deakin.edu.au (S.R.J.); dan.dias@deakin.edu.au (D.A.D.); 2 Deakin Centre for Advanced Food Sciences, School of Exercise and Nutrition Sciences, Deakin University, 221 Burwood Highway, Burwood, VIC 3125, Australia; 3 Noumi Pty Ltd., Shepparton, VIC 3630, Australia

**Keywords:** dairy flavour, UHT milk, flavour analysis, volatile compounds, analytical methods

## Abstract

Milk is a globally important food valued for its nutritional content and accessibility, with extended-shelf-life products such as ultra-high-temperature (UHT) milk increasing market reach but often altering flavour and impacting consumer acceptance. Despite the central role of aroma in determining flavour, analytical approaches in dairy research are frequently applied in isolation, limiting mechanistic insight into how processing-driven changes in volatile compounds influence sensory perception. This review critically examines analytical strategies for characterising aroma in bovine drinking milk, with emphasis on sample preparation, volatile extraction, chromatographic profiling, and sensory methodologies. Across the literature, inconsistent experimental design, limited consideration of aroma-active compounds, and the separation of chemical and sensory analyses emerge as key constraints. Evidence indicates that no single analytical approach is universally optimal, with method performance dependent on matrix composition and analytical objectives; however, multidimensional chromatography and integrated sensory–instrumental approaches provide clear advantages for resolving complex flavour systems. This review highlights the need for standardised, matrix-appropriate methodologies and demonstrates that improved integration of chemical and sensory data is essential for advancing mechanistic, consumer-relevant flavour characterisation in milk.

## 1. Introduction

Milk is a globally consumed food valued for its nutritional value, accessibility, and versatility in both fresh and processed products. As dairy markets increasingly rely on products with a longer shelf life such as pasteurised and ultra-high-temperature (UHT) milk, flavour has become a critical determinant of consumer acceptance. Although processing is essential for ensuring safety and stability, it can modify flavour characteristics in ways that influence product perception and commercial success.

Flavour perception arises from the integration of taste, texture, and aroma, with aroma recognised as the dominant contributor to overall flavour, accounting for approximately 75–90% of the sensory experience of food products [1]. In milk, aroma is governed by a complex mixture of volatile organic compounds (VOCs), many of which are present at trace concentrations but exert disproportionate influence due to low sensory thresholds. As a result, relatively small changes in volatile composition can lead to perceptible differences in flavour.

Milk VOCs originate from multiple biological and technological sources. In raw milk, volatile composition reflects animal diet, seasonal variation, and metabolic processes, forming a baseline chemical fingerprint that influences downstream products [2,3]. Processing then modifies this baseline through pathways such as lipid oxidation, Maillard reactions, and protein degradation, generating both desirable and undesirable aroma compounds [4]. Emerging or alternative processing approaches, including non-thermal technologies, may modify aroma via different mechanistic routes, with implications for both flavour and shelf-life [5]. Following processing, milk continues to evolve during storage, driven primarily by oxidative and degradative reactions that progressively influence aroma and consumer acceptance [6].

Despite the recognised importance of aroma on consumer perception, robust characterisation of milk volatiles remains challenging. Milk is a complex matrix with high water content and substantial lipid and protein fractions that can suppress, bind, or transform volatile compounds, complicating their extraction and analysis. While gas chromatography (GC)-based approaches are widely applied, variability in sample preparation, separation conditions, and detection strategies has contributed to inconsistent reporting and limited comparability across studies. In addition, many studies emphasise total volatile profiles rather than identifying aroma-active compounds that drive sensory perception [4]. Chemical and sensory datasets are also frequently generated in parallel but interpreted independently, limiting mechanistic insight into how specific chemical changes translate into perceived flavour differences. When sensory measurements are included, they are often limited to screening approaches using electronic sensor arrays, further constraining interpretation from the compound to the perception level [5,7,8]. Together, these factors have limited a coherent, mechanistic understanding of how processing and storage alter aroma in drinking milk and how analytical choices shape the conclusions drawn.

Previous reviews have discussed dairy flavour chemistry and volatile analysis more broadly, including work on specific origins of flavour and off-flavours in dairy [4], methods for volatile profiling of general food matrices [9], and processing methods used for milk [10]. However, there remains a need for a critical, method-centred review that (1) focuses specifically on bovine drinking milk as a matrix, (2) evaluates how methodological decisions in sampling and analysis influence the VOCs reported, and (3) explicitly links instrumental output with sensory relevance through aroma activity and integrated sensory-instrumental study designs.

As such, the objective of this review is to critically examine analytical approaches for characterising aroma compounds in bovine drinking milk, with emphasis on sample preparation, volatile profiling techniques, and sensory methodologies. By identifying key methodological limitations, clarifying best-practice considerations, and highlighting opportunities for improved sensory instrumental integration, this review aims to support more mechanistic and consumer-focused flavour characterisation. Particular attention is given to integrated, multi-layered analytical strategies, including emerging multi-omics and advanced data analysis approaches, that have the potential to better resolve the chemical drivers of flavour change in complex dairy systems.

## 2. Methodology

A literature review was conducted using the EBSCO database to identify articles. The following criteria were used to screen and select articles: (1) studies involving bovine dairy milk, (2) studies that focus on analytical chemistry techniques for the analysis of volatiles, and (3) sensory data used as supporting information for instrumental approaches. The search strategy for this review involved using keywords such as “dairy”, “milk”, “volatile*”, “aroma”, “flavo*”, “analytical chemistry”, “GC—MS”, “GC × GC”, “SPME” and “olfacto*”. Studies were included in this review if they were published in the last 15 years and were peer-reviewed. Studies were excluded if they focused on other animal milks (such as sheep, horse, or human milk) or fermented dairy products, or if they relied exclusively on sensory methods without instrumental support. Some studies are included as exceptions to the exclusion criteria because of their high applicability.

## 3. Chemical Drivers of Milk Aroma

The flavour of cow milk is derived from the holistic experience of physical properties, non-volatile taste compounds and volatile aroma compounds. A wide range of tastes have been studied in milk, with sweet and buttery flavours being common positive flavours, while sour, rancid, and cooked flavours are associated with negative consumer experiences [11]. The following sections will focus on the aroma of milk and its impact on milk flavour. The various origins of key aroma compound classes will be discussed, from the impact of feed and season to processing effects and finally to long-term chemical reactions that can influence the profiles.

### 3.1. Volatiles in Raw Milk

Volatiles in milk can originate from a range of sources, with various environmental and processing factors contributing to their formation. However, it is important to identify volatiles originating in raw, unprocessed milk and the factors that can influence the starting chemical fingerprint before processing, as the compounds in raw milk may have an ongoing impact on the flavour of processed milk. Previous reviews have provided lists of aroma compounds in various milk matrices, with raw milk typically containing the fewest identified VOCs [4]. Initial investigation by Moid et al. (1994) into the aroma profile of raw milk identified two esters (ethyl hexanoate and ethyl butyrate), dimethyl sulphone and nonanal, 1-octen-3-ol and indole, indicative of fruity, green, fatty, musty, and heated milk aromas [12]. While advances in separation science have improved sensitivity, direct sensory analysis via olfactometry has not been conducted, preventing a direct comparison between early aroma profiles in raw milk and more recent advances. However, recent studies have begun to investigate how different factors in cattle rearing can affect the volatile profile and, therefore, the flavour of milk.

Feed type has been investigated as a possible origin of many volatile compounds that can contribute to flavour. Faulkner et al. (2018) compared pasture and mixed-feed diets and found that the feed type significantly affected aroma-active volatiles in bovine milk, with grassy, cowy, and barnyard flavours more prevalent in pasture-raised milk and more sweet, malty flavours in mixed-grain milk [13]. Additional research by Zacometti et al. (2023) has investigated how different mixed feeds affect milk volatiles, comparing silage and dry-stored hay [3]. This investigation found that the type of mixed feed did not significantly influence the volatiles present in milk. However, the authors found that seasonal variation in milking significantly affected volatile levels [3]. It was determined that milk collected in winter contained higher concentrations of carboxylic acids, whereas summer milk showed higher abundances of two methyl aldehydes and 2-pentanol. While a direct causal link cannot be identified, it was proposed, supported by other studies, that incomplete esterification of carboxylic acids during winter led to increased acid content [2]. It was posited that the aldehydes present in the summer milk, rather than being an oxidation product, were amino acid degradation products, likely formed in the feed during storage [14]. 2-Pentanol was suggested to have originated from early lipolysis and oxidation or to be a metabolic product of microbial populations in the milk [13,15]. This work underscores the importance of seasonal control in milk research, as raw milk volatiles are likely to influence subsequent changes during processing.

### 3.2. Volatiles from Pasteurisation

Processing-induced changes can result from thermal and non-thermal processing of milk. Thermal processing can lead to the formation of several desirable and undesirable flavours, usually attributed to Maillard reactions catalysed by the increased thermal loads used in pasteurisation. While they may cause undesirable flavours to develop in the milk, these thermal processes are necessary to eliminate harmful microbial species from raw milk, making it safe for consumption. The thermal load degrades milk proteins into sulphur-containing amino acids, which react with natural sugars to form a range of Maillard products. Secondary to these, Maillard products are sulphur-containing compounds that can impart eggy and burnt off-flavours. The Maillard products encompass a range of aroma-active volatiles, including furfurals and furanones (characterised by sweet, caramel, and fruity aromas), pyrazines (associated with roasted flavours), pyrroles (caramel or heated plastic aromas), and thiazoles (noted for popcorn or sulphurous aromas) [16]. Strecker aldehydes are intermediates in the Maillard reaction and can form in milk products, imparting sweaty, malty, or fruity aromas [17]. Pasteurisation of raw milk is the mildest form of commercial thermal processing, in which the milk is heated to 72 °C and held at that temperature for 15 s. This approach, known as high-temperature short-time (HTST), is used to kill the most common microbial species in raw milk, thereby extending shelf life and reducing the risk of foodborne illness. While still a mild treatment method, pasteurisation does result in some changes in the milk’s volatile compounds. In a separate assessment of pasteurised milk, eight additional aroma-active volatiles were reported relative to raw milk, alongside the disappearance of certain VOCs and changes in the relative abundance of compounds already present [12]. The aroma profile was dominated by dimethyl sulphone and hexanal, with the sulphone content being significantly higher than in raw milk [12]. Negative aromas are more common in pasteurised milk, with a burnt-rubber aroma developing and musty aromas increasing in raw milk. However, there were additional positive aromas, with fruity, floral, and sweet notes associated with increased ketone levels.

An alternative to the common HTST processing is batch pasteurisation, in which the milk is heated to milder temperatures of 30–60 °C for 10–50 min [8]. Toelstede and Hofmann (2008) investigated the impact of these temperature and time conditions on the volatile profile and consumer perception of skim milk, showing that temperature affected only the total content of acids, aldehydes, and ketones [18]. Sensory analysis, through rating on a 9-point hedonic scale, showed that 50 °C gave the highest scores for positive sensory properties, including “milk flavour”, “butter”, and “aroma”, while having the lowest score for “off-flavour”. The acid and aldehyde content of the 50 °C sample was highest. The individual acids were relatively low in concentration, likely imparting a buttery aroma while also serving as precursors for other aroma compounds [18]. Similarly, the individual aldehydes present at low concentrations imparted fresh, green aromas but could indicate off-aromas at high concentrations [19]. A 30 min hold time was found to be associated with the same sensory outcomes, with different volatile outcomes. The 30 min hold time showed the lowest concentrations of ketones, aldehydes and alkanes, whilst having the highest acid content. Again, individual acids were present in low concentrations, resulting in pleasant aromas. Among the volatile classes examined, ketones are of particular interest, as their formation was dependent on processing time rather than temperature. Ketones are associated with desirable aromas, whereas the absence of ketones is primarily linked to fat loss [20]. Pasteurisation represents the mildest range of changes to the volatiles and, therefore, aroma of milk for commercial consumption. The fresh, green aromas and slight sulphurous notes of raw milk are still present; however, the addition of Maillard products alters the profile in detectable ways. Namely, the increasing number of Strecker aldehydes leads to more cooked, malty and sweaty notes that can impact consumer acceptance. Additionally, the ketone content increases in pasteurised milk, promoting floral and sweet aromas within these processed milks. While there are key changes in the aroma and volatile profiles of pasteurised milk, it remains a mild thermal treatment. The higher thermal load of UHT treatment may have a more significant impact on the milk.

### 3.3. Volatiles from UHT Treatment

**Figure 1:**
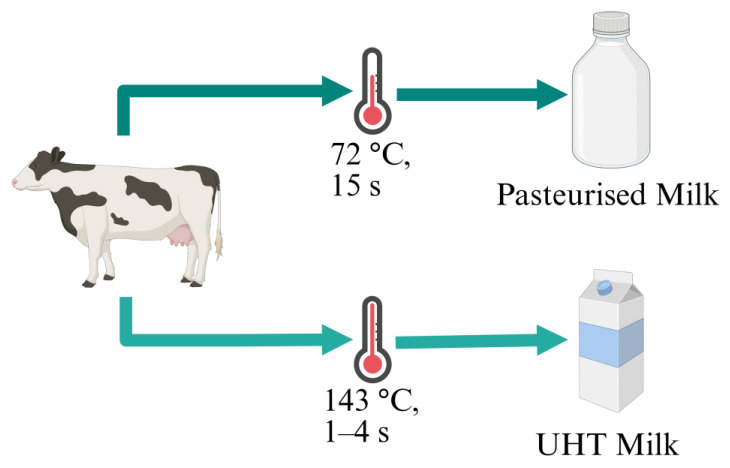
Thermal Processing Techniques for Bovine Drinking Milk. Created in BioRender. FRC, C. (2026) https://BioRender.com/1o9dhf7, Accessed on 24 May 2026.

UHT milk is a long-life product, viable for consumption months after processing because it eliminates most pathogenic and spoilage microorganisms from raw milk [21]. Complete sterilisation of these products requires an intense thermal load far higher than that of traditional pasteurisation. Two types of UHT sterilisation are used in commercial processing systems: indirect UHT (IND-UHT) and direct steam injection (DSI-UHT). DSI-UHT involves direct exposure of the milk to steam at 143 °C, with the steam remaining in contact with the milk for 1–2 s. IND-UHT uses steam to heat plates to 143 °C, after which the milk is passed through them, remaining in contact with the plates for 3–4 s [22]. Figure 1 below shows the standard temperatures and holding times used for traditional pasteurisation and UHT processing of bovine milk.

Comparison of raw milk, HTST pasteurised milk, IND-UHT and DSI-UHT showed that HTST has the closest volatile fingerprint to that of raw milk, agreeing with previous analyses [12,22]. When the two UHT treatments were compared, DSI-UHT exhibited an aroma profile that most closely resembled that of raw milk. This is likely due to the shorter steam-contact time, which reduces the thermal load and limits reaction progression within the milk matrix compared to IND UHT. While DSI-UHT more closely resembles the volatile profile of raw milk than IND-UHT, both are still significantly different from pasteurised and raw milk. In a study by Moid et al. (1994), analysis of aroma compounds in UHT milk showed a significant decrease in desirable fruity and grassy aromas, with an increase in burnt, cooked and vegetal aromas [12]. The aroma profile of UHT milk was dominated by off-flavours associated with Maillard products and degradative thermal reactions [4]. Another study identified a broader range of aroma-active compounds present in UHT milk [23]. Czerny and Schieberle (2007) identified a range of lactones, alcohols, aldehydes and acids in both types [23]. The aroma compounds represent several desirable aromas, including buttery, fruity, roasted, and vanillic aromas; however, the majority of aromas presented undesirable notes, including rancid, burnt, sweaty, musty, and cardboard. Comparison of the UHT and pasteurised volatile profiles showed that UHT milk had a lower abundance of aldehydes, alcohols, sulphur compounds, and aromatic and heterocyclic compounds [22]. The esters and acids in the DSI-UHT were found to be similar in content to the other milk matrices, but acid content was much higher and ester content much lower in IND-UHT. Ding et al. (2023) proposed that aromatic and heterocyclic compounds likely underwent reactions catalysed by the high thermal stress of UHT processing, leading to breakdown and conversion into other volatile constituents [22]; however, the possible mechanism was not discussed. The sulphur-containing compounds likely underwent a similar process, with the increased exposure time of IND-UHT resulting in the lowest sulphuric content due to thermal degradation. Similarly, alcohols are common precursors to other aroma compounds and would have been converted into them at the required temperatures. Aldehydes are produced through Strecker degradation in milk, which is associated with fatty acid metabolism by bacterial species in milk [24]. The low aldehyde content in UHT milk was linked to the bactericidal capacity of UHT processing, as the reduced bacterial load limited metabolic processes that produce these aldehydes. The acids are typically derived from the hydrolysis of fatty acids, enzymatic catalysis, or the thermal degradation of lactose and amino acids during Maillard reactions [25,26]. The thermal load of UHT processing would have exacerbated these reactions, resulting in higher acid content in UHT milk. DSI-UHT had an acid content similar to that of the other milk matrices. At the same time, IND-UHT was significantly higher, indicating that exposure to higher temperatures for longer times further catalysed these reactions. The ester content was linked to the metabolism of fatty acids in milk by microorganisms, with milder pasteurisation and DSI-UHT conditions leading to increased ester content. Finally, ketones were detected only in the 85 °C HTST and UHT processes, with IND-UHT showing the highest concentration. The detected ketones are formed by thermally induced decarboxylation and oxidation of acids, so that the higher thermal load leads to a greater abundance of ketone species [27]. Overall, UHT processing led to a more complex volatile and aroma profile, dominated by undesirable off-aromas via Maillard reactions. The key changes observed in UHT milk products are an increase in cooked, stale and burnt notes, linked to the aldehydes and sulphur compounds produced by the Maillard reactions facilitated by higher temperatures. The acid content of these UHT milks is also higher than that of pasteurised milk, giving rise to more detectable sweaty and cheesy notes. The production of safe-to-drink milk is typically achieved through thermal processing, but other methods can achieve the same goal without thermal degradation.

### 3.4. Volatiles from Other Processing Methods

Three major non-thermal processing techniques have received attention in the literature, namely ultrasonic processing, Ultraviolet light–C (UV–C) and high hydrostatic pressure (HHP) processing. HHP has been widely applied in dairy research, with available comprehensive reviews outlining its principles and applications [28]. A brief discussion of the technique’s effect on the volatile flavour compounds in milk is presented here. HHP alone has been shown to modify the volatile profile of milk, significantly increasing the formation of volatile compounds in milk [29]. However, the addition of a transglutaminase enzyme, which can prevent degradative reactions in milk matrices, did not affect the formation of volatiles under HHP treatment. In addition to standalone HHP, the combination of pressure and thermal processing has been investigated in both human and bovine milk. In bovine milk, aldehydes, ketones and sulphur compounds increased in abundance after processing [30]. The affected volatiles are indicative of Maillard products, suggesting that the combination of pressure and thermal processing could not preserve the aroma of bovine milk. This work conducted a broad analysis of the effects of temperature, time, and pressure on the volatilome, finding that increases in volatile abundance were positively correlated with processing time and temperature. This aligns with the increasing trend toward greater volatile complexity in thermally processed milk compared to raw milk. Dynamic HHP, in which the milk was exposed to changing pressure conditions, showed that pressure-based processing affected the volatilome, with different dynamic processing methods yielding higher levels of aliphatic hydrocarbons and carboxylic acids, whilst also showing lower levels of aromatic hydrocarbons than in raw milk [5]. Overall, HHP may be beneficial as a non-thermal processing technique for milk; however, some volatile changes may still occur during processing. While sensory analysis has not been conducted for these HHP milk products, the increased aldehyde, ketone, and sulphur content would have similar effects on the overall aroma as observed with thermal processing techniques. There would likely be increased levels of burnt and cooked notes associated with aldehydes, with ketones imparting various aromas depending on the specific ketones formed. However, as these compounds are typically associated with thermal processing, the overall aroma impact will likely be less than that detected in pasteurised and UHT milks.

UV–C processing has been investigated for bovine milk. When sensory analysis was conducted on milk for cheese production, with pasteurised milk as a control, a similar trend was observed, with pasteurised milk showing significantly better sensory outcomes than UV-processed milk [31]. Volatile analysis of UV–C milk showed an increase in the abundance of aldehydes, alcohols, aliphatic hydrocarbons, esters and ketones when compared to HHP and pasteurised milk [5]. The specific mechanism of their formation is unknown, but increased oxygen availability in UV–C processing may have facilitated oxidation reactions within the matrix. Additionally, the formation of aldehydes is known to be accelerated by light-mediated lipid oxidation in milk matrices [30]. The more complex volatile profile of UV–C processed milk will impart a range of aromas to the final product, indicating a need for comprehensive sensory analysis. The possible link to lipid oxidation will likely promote off-aromas, including burnt, cardboard and stale notes linked to increased aldehyde and ketone content.

Ultrasonic processing is one of the most popular non-thermal processing techniques for bovine milk. It is widely used for its ability to improve processing efficiency and maintain the functional properties of milk products. While limiting milk exposure to high thermal loads, ultrasonic processing does induce some volatile changes, mostly linked to oxidation reactions driven by free radicals generated by ultrasonic disruption of microorganisms within the matrix [32]. Cheng et al. (2025) investigated how high-intensity ultrasonication affected the interactions between volatiles and β-lactoglobulin, which is known to bind various chemical compounds and possibly impact flavour [33]. This investigation indicated that β-lactoglobulin can bind volatile compounds, affecting aroma retention in milk, and that ultrasonic treatment alters the binding mechanism, leading to a higher number of ketones within the matrix [33]. However, this work did not determine whether these were naturally occurring ketones in the milk or oxidation products from ultrasonic processing. Chouliara et al. (2010) investigated how ultrasonic processing interacted with different thermal processing methods and the resulting impact on microbial, chemical and sensory properties [34]. The investigation found that ultrasonic treatment resulted in taste scores equal to or lower than those of untreated milk for all milk samples [34]. When considering lipid oxidation factors, it was determined that ultrasonication did significantly increase the rate of lipid oxidation; however, it was still lower than the limit at which it becomes sensorially unacceptable [35]. Additionally, it was determined that secondary oxidation products, namely aldehydes, increased during storage in ultrasonicated samples, whilst aldehyde abundances remained relatively stable in untreated milks [34]. These investigations indicate that while ultrasonic processing may be an effective non-thermal processing technique, the sensory and volatile impact may be a limiting factor for consumer acceptance. While these novel processing techniques have shown promise, additional evidence is needed to clarify their impacts on volatile profiles and sensory outcomes before large-scale implementation.

### 3.5. Volatiles in Lactose-Free Milk

Lactose-hydrolysed, or lactose-free, milk is a key product to consider given the rising prevalence of lactose intolerance in consumers. While these products are becoming increasingly popular, research into the volatile changes in these milks remains limited. Jansson et al. (2014) investigated the volatiles present in UHT-processed lactose-free milk and compared them with those in conventional UHT and filtered (low-lactose) UHT milk [36]. This study revealed that conventional UHT milk had the highest ketone abundance, which is responsible for stale notes in UHT milk [36]. Aldehydes and ketones, as products of the Maillard reactions, are dependent on the sugar content within the milk matrix during processing. The removal of lactose from the milk will likely limit these reactions, resulting in the decreased ketone concentration observed in the lactose-free products. The reduced ketone content is supported by Jensen et al. (2015), who compared conventional UHT milk to lactose-free milk processed using direct and indirect UHT technologies [37]. This study revealed the importance of UHT processing technologies: direct UHT led to an increase in ketone abundance in lactose-free milk during storage, whereas indirect UHT started with a higher ketone abundance that continued to decrease during storage [37]. This investigation revealed that lactose-free milk, regardless of direct or indirect UHT, had a higher bitterness intensity than conventional UHT milk, linked to higher levels of primary amines resulting from proteolytic reactions. The higher bitterness in lactose-free milk was linked to the activity of the β-galactosidase enzyme added to hydrolyse lactose, suggesting a need to identify more effective methods for removing lactose to maintain sensory outcomes.

### 3.6. Flavoured Milk Production

Another key processing application is the production of flavoured milk products. In 2019, 26.5% of the dairy and plant-based milk products sold in Australian grocery stores were flavoured [38]. During production, flavourings (e.g., chocolate and coffee) are mixed into raw milk before pasteurisation or UHT processing, with thermal processes, such as Maillard reactions, affecting the final flavour [39]. Concentrated flavour additives overshadow the subtle natural flavours of milk, making the aroma-active compounds of the flavouring more influential than those of the milk itself. Production typically begins with separating raw milk into cream and skim milk, followed by standardisation to achieve the desired fat content. Flavouring mixes and sugar are added before heat treatment, after which the milk is homogenised to ensure uniform fat distribution, then cooled and packaged [40]. Chocolate milk uses cocoa powder as a base for flavourings, producing an intense chocolate taste with minimal sedimentation [39]. Coffee-flavoured milk typically uses freeze-dried instant coffee [41], whereas fruit-flavoured varieties, such as strawberry and banana, rely on synthetic syrups [42]. The intensity of aroma compounds in these flavourings largely determines the final aroma profile. The production of these flavoured milk drinks requires careful balancing, as excessive flavour addition can negatively affect consumer liking [43,44]. These flavoured milk products, while holding significant market share, are not commonly analysed, indicating a large research gap. Flavoured milk products are understudied, with only one study using an analytical approach for flavour analysis. The impacts of flavoured milk on flavour stability and shelf life have not been investigated in the literature. Possible drivers of this research gap include intellectual property and trade secrets in manufacturing these flavoured products, as specific formulation information is closely guarded by manufacturers. Additionally, these products may be underinvestigated due to a perception that they are low-value in the market, prompting researchers to focus more on unflavoured products, which have a higher market share. The impact of processing on the flavour of milk is critical, but equally important are the reactions that occur during storage and affect these outcomes.

### 3.7. Aroma Evolution During Storage

The key reactions of interest when studying longitudinal changes in milk during storage are lipid oxidation. Lipid oxidation is a dominant mechanism underlying flavour deterioration in milk during storage and a major contributor to shelf-life limitation. Thermal processing can initiate oxidative reactions by inducing lipid radical formation and altering the milk matrix, after which auto-oxidative pathways continue to propagate during storage. The ongoing formation of aldehydes and ketones through these reactions is closely linked to the emergence of off-flavours and declining consumer acceptance [4]. These continuous reactions can lead to sensory rejection, as the continuous formation of undesirable aromas will deteriorate the sensory profile during storage.

The impact of storage on milk flavour has been extensively studied, with a focus on various milk types and the mechanisms underlying these changes. Raw milk, while representative of the base flavour, is susceptible to shifts in its volatile profile during storage, which can negatively affect flavour. Raw milk stored on farms under refrigeration for several days before processing may exhibit changes. Microbial activity will primarily be responsible for changes in raw milk volatiles, thereby compromising quality before thermal processing [45]. In pasteurised milk, storage significantly alters the volatile profile. Karatapanis et al. (2006) conducted a 7-day storage trial, the results of which showed increases in dimethyl sulphide and aldehydes, suggesting that light-induced oxidation, autoxidation, and microbial oxidation can affect flavour [6]. Additionally, the fatty acid composition of pasteurised milk contributed to the formation of acetic acid, ethyl esters, and aldehydes, imparting sour and cooked notes, even when stored at cold temperatures [46]. In contrast, Yan et al. (2024) showed that UHT milk had greater stability, with no significant changes in the volatile profile during the first 90 days of storage at 25 °C [46]. However, Maillard and proteolytic reactions began to alter flavour after this period. These volatile shifts were linked to a decline in sensory acceptance, highlighting their influence on overall flavour [47]. However, these studies have not exceeded four months of storage, indicating a lack of research on the total shelf life of UHT milk, which is typically 9–12 months. In flavoured milk, cold storage was unable to prevent volatile changes, some of which were associated with the development of undesirable aromas and the loss of positive ones [48].

Another key factor driving volatility during storage is the specific packaging used to bottle the milk. Simon and Hansen (2001) evaluated how different packaging types impacted the taste of milk refrigerated for 15 weeks [49]. Standard cardboard packaging had a higher rate of flavour deterioration than packaging with a foil barrier [49]. Additionally, cardboard packaging imparted a notable cardboard flavour, while the foil barrier imparted a more pronounced cooked flavour. The cardboard flavour continued to intensify, while the cooked flavour deteriorated. Karatapanis et al. (2006) investigated a wider range of packaging materials, including clear glass, clear PET, coloured PET, three-layer co-extruded HDPE, coloured monolayer HDPE and cardboard [6]. The analysis revealed that clear packaging exhibited higher volatility, whereas coloured packaging reduced the observed rate of change [6]. This is likely due to the amount of light being blocked by the coloured packaging, reducing the influence of photo-oxidation, as indicated by the absence of dimethyl sulphide, a key photo-oxidative product, in milk stored in darkly coloured bottles. The selection of appropriate packaging materials will affect the volatile profiles during storage and the sensory acceptance of these products. The range of products assessed, from raw milk to flavoured UHT milk, showed that long after thermal processing, milk products continued to change. However, to completely understand the volatile and aroma changes, appropriate analytical techniques and sensory analysis methods are needed. The complex methods available are presented in the following sections.

## 4. Sample Preparation Methods

To effectively analyse the volatile profile of milk, sample cleanup procedures are necessary. The high lipid and protein content of milk can interfere with instrumental analysis; therefore, effective extraction strategies are required to isolate volatile compounds from the matrix. These extraction techniques can often be differentiated by the part of the sample in which they are applied: volatile headspace or direct liquid extraction. Table 1 summarises selected sample preparation methods. As indicated, headspace-based methods were most popular, with five approaches assessed across 18 experiments. Solvent extractions were the second-most-preferred approach, with five experiments in total. Liquid-immersion techniques were the least popular, with only one experiment identified. The following sections will address headspace and liquid methods (including solvent and immersion) in more detail.

### 4.1. Headspace Techniques

Various headspace extraction techniques have been investigated for extracting volatiles from milk. By extracting volatiles from the milk headspace, less matrix interference is required. Headspace methods are less prone to interference from milk’s lipid and protein content. The primary consideration in headspace extraction is the ability of lipids to trap certain volatiles, which can lead to inconsistent volatile profiling.

#### 4.1.1. Solid-Phase Microextraction

Among headspace extraction techniques, headspace solid-phase microextraction (HS-SPME) is the most widely used. The popularity of this method stems from the convenience of the SPME approach, which is well-suited to both targeted and untargeted analytical methods.

##### SPME Optimisation

Studies employing SPME methodologies vary widely. A consistent approach is lacking, with different groups using a wide range of fibre coatings, equilibration times and temperatures, extraction conditions, and desorption settings before injection on an analytical platform. The absence of a formal optimisation study in milk means researchers often rely on previously published conditions or personal experience. In recent studies on milk that mention optimisation, there is often no structured design of experiments (DOE). These optimisations typically start with an arbitrarily chosen temperature, at which three time points are tested. The best time is then selected, followed by three additional trials at different temperatures. There is little consideration of interactions between factors, making these optimisation efforts statistically ineffective. Despite limited cross-factor analysis, these exploratory studies may still reveal trends in temperature and time that inform the optimal analysis of milk volatiles. The equilibration and extraction temperatures for milk volatiles need to be carefully selected to balance preparation time, sample degradation, and artefact formation.

##### Temperature Considerations for SPME

Temperatures above 50 °C can catalyse degradative reactions in the milk matrix, leading to artefact formation and degradation of native volatiles. This can confound results and misrepresent the volatilome and flavour of the milk. Additionally, higher temperatures may promote the release of volatiles from the sample, but they do not necessarily increase the adsorption capacity of SPME fibres [57]. Reports by Hu et al. (2017), Pan et al. (2019), and Yue et al. (2015) used 50 °C as the equilibration and extraction temperatures [5,8,20], while Genovese et al. (2019) used 55 °C [52] and Jin et al. (2025) used 60 °C [53], the highest temperature reported. Conversely, low temperatures require longer equilibration and extraction times, which can lead to overly competitive adsorption by the more volatile species, preventing adsorption of heavier lactones and other semi-volatiles present in milk. In the included literature, the lowest temperatures were 20 °C [22], 18 °C [54], and 10 °C [51]. The remaining studies used temperatures of 35–45 °C, close to human body temperature. This temperature range may be more advantageous for analysing food products, as it maximises the extraction of volatiles whilst limiting thermal degradation during extraction procedures. In addition, temperature considerations indicate that the time at temperature can significantly affect the volatiles extracted from milk.

##### Equilibration Time

When planning HS-SPME analysis, two major time points must be accounted for: equilibration and extraction. Equilibration refers to the period during which samples are heated to allow volatiles to enter the headspace and form a stable equilibrium. This allows the initial extraction to proceed and heavier volatiles to migrate into the headspace for extraction. Not all studies included an equilibration step, which may reduce the sensitivity of the extraction method. In a study by Tufariello et al. (2019) on wine, it was shown that the equilibration time of an HS-SPME method significantly affects the total peak area extracted [58]. As such, the inclusion of an equilibration step may be crucial for improving the sensitivity of HS-SPME in volatile analysis of milk. When including an equilibration step, it remains important to consider the duration of extraction and analysis, particularly when high-throughput analysis is required. In wine, an equilibration step of 30 min or more has a significant impact, suggesting that times around 30 min should also be tested for milk, given the aqueous sample matrix. However, among the studies targeting approximately 30 min, only Danesi et al. (2024) and Rashid et al. (2019) included an appropriate equilibration time [51,54]. It is important to note that these are the two papers that used the lowest temperature, likely overcoming poor volatilisation at low temperatures by using a longer equilibration [51,54].

##### Extraction Time

In the same wine analysis, extraction time was positively linearly correlated with total peak area; however, this correlation was not statistically significant [58]. The level of significance was determined within a 30 min time constraint, indicating a quadratic relationship; thus, extraction times beyond 30 min may have a greater effect. While the effects of extraction time have not been fully studied, it has been reported that extraction times exceeding 60 min are required to recover some compounds, such as heptanal, 3-heptanone, 2-heptanone, and 2-pentylfuran [54]. This was determined in a study by Rashid et al. (2019), who used an 18 °C extraction temperature and a short 18 min equilibration, which may have contributed to the need for a longer extraction step for these compounds [54]. Maximising the number of extracted compounds is important for comprehensive profiling, and so any modifications that include more compounds are beneficial. For higher-temperature extractions, a shorter 30 min extraction step yielded the same result. The other cold extractions were conducted at 10 °C for 180 min and at 20 °C for 30 min. The longer extraction detected heptanal in the milk, whereas the 30 min extraction did not. A lack of heptanal may have caused this, as this study used UHT milk rather than raw or pasteurised milk. However, the trend suggested that, at low temperatures, longer extraction times are required for some of these compounds. When conducted at 50 °C, a 30 min extraction time proved sufficient to extract heptanal [20]. This indicated that a balance between temperature and time was necessary to ensure timely and efficient extraction. This further supports the idea that a full DOE was necessary to determine how extraction factors interact to improve the effectiveness of HS-SPME methods.

##### Sample Volume

Another significant source of variation was the sample volume used across studies, with 10 mL the most common, typically drawn from a 20 mL vial. As an upper outlier, a study used 22.5 g of milk in a 50 mL headspace vial, which is more than twice the most common volume [52]. At the lower end, two studies used 5 mL volumes in unreported-sized vials [3,53]. The volume used was 4 mL of coffee milk, again in an undisclosed-sized vial [48]. While there is significant variation in the total milk volumes used, no study has fully evaluated the effect of sample volume on HS-SPME capability in milk. However, other food types have been evaluated in a specific study on virgin olive oil, which showed that volatile adsorption increased with sample volume up to a plateau [59]. It was expected that milk would show a similar relationship, albeit possibly with a higher sample volume required before reaching the plateau. While larger volumes show greater adsorption capacity, this relationship may also depend on the available headspace volume in the sample vials. Larger sample volumes in smaller vials may have two negative effects: first, limiting gas–liquid partitioning due to limited headspace volume, and secondly, increasing the risk of wetting the fibre during agitation.

##### Addition of Salt

An additional factor to consider in SPME analysis is the addition of salt to the sample to reduce the analyte partition coefficient and promote analyte volatilisation. While not assessed directly in milk, the addition of anhydrous NaCl has been shown to increase the total number of extracted compounds from food products [60]. In the included studies, the amount of salt added to the samples varied, with some studies omitting it entirely. In three studies, a total salt content of 37–40% was added, representing the most common sample-to-salt ratio [5,20,53]. The highest amount added was 3.5 g of salt per 5 mL of milk, representing 70% of the volume of milk as salt [3], whilst the low end was 1 g of salt per 10 mL of milk. [22]. Until a full comparative analysis of the effect of salt content on volatile adsorption is conducted, an optimal salt content cannot be recommended; however, the literature trend indicates that an approximate content of 37–40% anhydrous NaCl is common for increasing volatilisation in milk [57].

##### SPME Fibre Coatings

The final consideration when optimising SPME for the analysis of milk volatiles is the specific phase used to coat the fibre. Different fibre coatings are selected for their affinity for specific analyte groups, and combinations of phases provide broad selectivity. Other reviews that focus more specifically on SPME technologies discuss the various fibre coatings in detail; therefore, a detailed discussion will not be presented here [61]. Polydimethylsiloxane (PDMS) is the most commonly used sorbent in sorptive extraction, as it enables extraction of non-polar analytes at moderate temperatures [62]. This makes it a broadly suitable phase for analysing volatile compounds in food products. When considering extractions conducted in milk analyses, the most common fibre coating is a triphasic coating of divinylbenzene/carboxen/polydimethylsiloxane (DVB/CAR/PDMS) [Table 1]. This phase combination enables the extraction of non-polar analytes at moderate temperatures, with increased selectivity for hydrocarbons due to the addition of the carboxen component, and higher selectivity for volatile compound classes such as alcohols, ketones, aldehydes, and nitrogen-containing compounds due to the divinylbenzene [61]. The next most common combination is a DVB/PDMS fibre coating, with two studies by Pan et al. (2019) and Rashid et al. (2019) using this fibre for analysis, which provides increased selectivity for the most common aroma compounds in milk products [8,54]. Another study used a CAR/PDMS fibre coating, which provided broader selectivity for hydrocarbons in comparison with a simple PDMS coating, but it did not focus on key aroma compound classes [51]. For a non-target analysis focused on aroma (and therefore flavour) in milk, the triphasic fibre offers the broadest selectivity for extracting a range of volatiles, especially key aroma volatiles. When a triphasic coating is not used, the next preferred option is the DVB/PDMS combination, as it enhances the extraction of aroma compounds with the broadest impact on flavour. In addition to the specific phase used, the type of SPME device used for extraction should be considered. All studies included in this review that used HS-SPME employed a traditional fibre, a solid core with an external coating sheathed in a protective needle. However, an increasingly popular alternative is the SPME arrow, which has a much larger sorptive surface area. This enables greater sensitivity, as there is less competition for the limited number of adsorption sites on a standard fibre [63]. The SPME needle also features a septum-piercing tip and increased external diameters of the support tubing and needle, which reduces the risk of mechanical breakage [64].

The optimum conditions for the extraction of volatiles from liquid bovine milk using HS-SPME are provided in Table 2 below. These conditions reflect a combination of the factors involved in designing SPME methods, which can be further optimised for individual research protocols moving forward. The optimum temperature is selected to maximise volatile adsorption while limiting artefact formation at high temperatures. The equilibration and extraction times led to a more comprehensive extraction of volatiles from milk when conducted at temperatures within the advised range. The sample volume, while not experimentally confirmed in the literature, fits within the range of volumes used in the literature. This provides a starting point for further research to optimise the volume for maximal volatile extraction. When adding salt, anhydrous NaCl is the standard salt, known to improve extraction efficiency in food products. The weight of salt added depends on the volume of milk used, representing 40% *w*/*v* salt in milk. This is again a starting point for optimisation experiments. Finally, for fibre coating, the PDMS/CAR/DVB fibre has been shown to extract the broadest range of volatiles from milk, making it the most suitable for analysing volatiles in milk. All recommendations are made assuming the goal is comprehensive volatile extraction, as targeted studies will need to be more focused on the desired analytes.

#### 4.1.2. Headspace Sorptive Extraction

HSSE offers another approach to sorptive extraction of volatiles from a headspace, relying on PDMS-coated stir bars for extraction and cryogenic desorption to transfer them to the GC. While not as popular for analysing milk volatiles, with only one study in this review applying the method, HSSE remains a powerful technique for volatile analysis [27]. By utilising headspace extraction methods such as SPME, HSSE bypasses matrix cleanup, reducing sample preparation time. Additionally, it can be conducted at moderate temperatures because of the PDMS coating, thereby preventing degradative effects during sample preparation. The HSSE approach may also be more sensitive than SPME due to a higher sorbent-area-to-sample-volume ratio. However, this may come with the drawback that PDMS is not particularly selective towards aroma compounds, which bi- and triphasic SPME coatings may better target [65,66]. HSSE also requires more specialised equipment than an SPME approach, including a thermal adsorption apparatus and cryogenic trapping to transfer volatiles from the stir bar to the analytical platform of choice. In a study by High et al. (2019), HSSE and SPME were compared for the determination of volatiles in spray-dried sheep’s milk [27]. The HSSE protocol used 8 g of reconstituted sheep’s milk in a 20 mL vial, heated in a water bath at 35 °C, with the stir bar suspended in the headspace. The extraction was conducted for 90 min with no equilibration step; however, this is appropriate for stir bar extractions, as adding the stir bar to the system would require uncapping the vial after equilibration, thereby losing volatiles. The low temperature and longer extraction time were selected to maximise sensitivity while limiting thermal load and preventing artefact formation, as determined in a series of unpublished trial experiments. The selected time and temperature are analogous to those previously used, with a temperature near human physiological standards and a longer extraction time to maximise sensitivity. Although the total extraction time remained longer than that of most SPME approaches, it may be necessary to compensate for the PDMS coating’s limited selectivity. Immediately after extraction, the stir bars were rinsed to remove any contaminants, after which the volatiles were thermally desorbed and cryotrapped. The cryogenic sample was then transferred directly into a GC–MS system for volatile profiling. This approach followed a structure similar to that of a previous analysis using HSSE to examine odour-active compounds in human breast milk, which used a standard approach [67].

For comparison, the SPME approach used the same temperature (35 °C) but a 60 min extraction, whereas HSSE used a 90 min extraction. The SPME fibre used had a triphasic coating; therefore, lower temperatures still enable the extraction of a broad range of volatiles. HSSE showed greater sensitivity, extracting 271 µg/kg of volatiles compared with 173 µg/kg for the SPME approach. This supports the hypothesis that a larger sorptive area would lead to greater extraction capacity, regardless of specific selectivity. However, the SPME fibre’s selectivity bias enabled it to extract dimethyl sulphide and pentanal, which were not detected by HSSE or other methods used in their study. Overall, SPME showed better selectivity for alcohols but was less effective at extracting lactones. SPME was also the most reproducible method, with a relative standard deviation (RSD) of 14.4%, compared with 26.7% for HSSE. Additionally, SPME showed the narrowest range of individual RSDs between replicates, with 7.0% for decane and 35.1% for dimethyl sulphone. In contrast, HSSE had the lowest RSD of 3% for 1-pentanol and a 99.5% RSD for acetic acid. An additional metric analysed by High et al. (2019) was the self-assessed efficiency of each method, measured by the time required to complete an extraction from setup to injection [27]. SPME outperforms the HSSE approach here, as fibres can be purchased pre-coated and do not require any special hardware beyond an autosampler (if using). By avoiding stir bar coating, post-extraction washing, thermal desorption, and cryogenic trapping, SPME reduces sample handling to simple vial preparation. However, as technology advances, sorptive extraction methods are becoming increasingly automated. This reduces hands-on time, as methods can be batched and executed sequentially using automated workflows.

#### 4.1.3. Headspace Sampling

The only other method of headspace sample preparation is direct headspace sampling. Headspace sampling can be performed using either a static or a dynamic approach. Examples of both have been included in this review: the static injection was used to analyse how different fatty acid ratios affect the volatilome of raw milk [55], and the dynamic approach was used to determine the impact of feed type on the aroma profiles of raw milk [7]. Both methods relied on collecting the sample headspace for direct injection into an analytical platform, thereby skipping sample preparation beyond delivering aliquots into an appropriate vessel. The difference between the two methods lies in how the headspace is collected for extraction. In static headspace sampling, samples are heated in a vial to drive volatiles into the headspace until equilibrium is reached. The headspace is then collected in a syringe or transferred directly to the analytical platform. Dynamic headspace uses a constant purge gas flow to sweep the sample headspace, carrying volatiles into an adsorbent trap for collection. The volatiles are then thermally desorbed and cryofocused for injection into the platform. Static headspace analysis has been applied to milk before, where it was found to be effective for analysing the most abundant volatiles, as the smaller trace quantities could not be effectively equilibrated [68]. When determining the impact of fatty acid ratios on volatiles in raw milk, static headspace was employed and proved viable for detecting differences between milk samples with variable fatty acid ratios [55]. Although effective, this approach used a high temperature of 80 °C with 20 min of equilibration. While this allowed for a short sample-preparation time, the high temperature likely caused degradative effects in the milk matrix, potentially leading to artefact formation and confounding volatile analysis. Previous work by Fabre et al. (2002) used 25 °C for 60 min, maintaining a moderate temperature, although it required a much longer equilibration step [68]. The movement of gas in a dynamic headspace approach, combined with a sorbent-trapping step, may allow the extraction of a broader range of volatiles, making it better suited to more complex samples. Caputo et al. (2015) analysed how different feeds affected the aroma profile of milk [7]. In this study, milk was heated to 40 °C and continuously extracted with nitrogen for 60 min. This moderate temperature would have accelerated the volatilisation of analytes whilst preventing artefact formation at high temperatures [7]. A direct comparison between the static and dynamic approaches cannot be made in these two studies; however, the approaches have been compared previously in their ability to extract volatiles from a standard test mix [69]. While not representative of the complex matrix of milk, this comparison still provides useful insight into the capacity for these methods to extract volatiles of interest. Static headspace sampling extracts significantly fewer volatile compounds than dynamic headspace sampling and shows reduced performance across other analytical metrics. Static sampling has an RSD of 8.8% across 41 analytes, whilst dynamic sampling has an RSD of 4.1%. When determining method detection limits for fatty acid analysis, static sampling had a limit of 45.0 ng/L, while dynamic sampling had a limit of 2.3 ng/L [55]. Overall, dynamic headspace analysis showed better performance across the board for volatile analysis.

While the headspace of milk is critical to flavour discussion, consideration must be given to heavier, semi-polar aroma compounds that may not be readily extracted from the headspace. Compounds such as lactones are frequently missed by methods that use short headspace sampling, as they remain within the milk’s liquid matrix. As such, various methods have been employed to extract volatile compounds of interest directly from milk, some examples of which will be discussed in detail in the next section.

### 4.2. Liquid Techniques

Solvent-based extraction of volatiles from milk can be performed using a wide range of methods, from sorptive extractions, such as SBSE, to targeted methods for fatty acid extraction. While less common than headspace preparation methods, liquid extraction can still be an effective method in analysing volatiles in milk, especially the heavier and more polar compounds.

#### 4.2.1. Solvent-Assisted Flavour Evaporation

The most popular method for extracting volatiles from milk was solvent-assisted flavour evaporation (SAFE). Although less common than conventional approaches, SAFE is a sensitive technique for capturing and preserving important aroma compounds directly from the sample [70]. SAFE uses highly specialised glassware, bulk solvent extraction, high-vacuum distillation, and cryo-trapping to extract a broad range of volatiles from both the liquid and headspace phase of a sample [71]. SAFE is conducted using dichloromethane or diethyl ether as a bulk solvent to facilitate the transfer of volatiles from the aqueous milk matrix into a suitable organic phase. The mixture is introduced into a sample-collection trap located above a delivery valve. The specialised SAFE glassware is evacuated to 10^−^^4^–10^−6^ mbar, with heated water warming the glassware’s body to facilitate volatilisation. A non-volatile collection vessel is submerged in heated water, and a volatile collection vessel is chilled in liquid nitrogen. Above the volatile collection vessel, a safety cold trap is chilled with liquid nitrogen to prevent thermal stress on the glassware. The sample is then slowly introduced by manually releasing the delivery valve, with 5 mL aliquots delivered into the glassware to facilitate vapourisation of volatiles and solvents [71].

##### Modifications to SAFE Methodology

While sample aliquots are commonly delivered into glassware via manual valve release, the SAFE apparatus has recently been modified to include a semi-automated pneumatic switching valve system [72]. This modification improves reproducibility by reducing human error in sample delivery.

##### Temperature Considerations for SAFE

In the two papers that applied SAFE to extract volatiles from milk, significant differences in methodology are evident. An initial point of difference was the temperature at which the SAFE apparatus was thermostatted: one group selected 35 °C [27] whilst the other selected 50 °C [22]. Both methods were conducted at a higher temperature than reported in the original paper, indicating that a temperature between 20 and 30 °C was appropriate [71]. This temperature was used for a model sample matrix, so higher temperatures may be necessary. This is supported by another study in which the authors used SAFE for dairy analysis. In their work, a temperature of 45 °C was used to facilitate the extraction [70]. The higher temperatures used in these studies may be selected to improve the recovery of semi-volatile components, reduce overall sample viscosity, and accelerate distillation. However, increasing the temperature may accelerate lipid oxidation during distillation, and pre-existing Maillard and sulphur compounds may be concentrated in the SAFE extract. This results in a sacrifice of authentic aroma fidelity in the samples to achieve total recovery. This is beneficial for studies focused on total volatile profiling; however, it prevents the determination of an authentic aroma profile. As such, the use of high temperatures in SAFE limits the technique’s effectiveness for true flavour analysis.

##### Sample Volume and Solvent Selection in SAFE

The next point of contention between the 2 studies concerns the volumes of milk and solvent used: 250 g of reconstituted dried sheep’s milk was combined with 100 mL of dichloromethane in one study [27], while 200 mL of UHT milk was mixed with an undisclosed volume of dichloromethane in the other [22]. While not an egregious difference in sample volume, this is representative of an ongoing lack of standardisation for methods employing SAFE. Both studies exceeded the volume of work previously conducted on bovine milk, which used 150 mL [70]. However, these are significantly lower than those reported in another commonly referenced study, which delivered 2 × 2 L aliquots into the sample trap [73]. The inconsistencies in SAFE methodologies are a major limiting factor for research seeking to employ the method, as there is no agreed-upon basis on which to work. This is further complicated by the addition of solvents, as there are significant differences in approach. While both Havemose et al. (2016) [70] and Bendall (2001) [73] added the solvent before distillation, as in the original protocol by Engel et al. (1999) [71], others complete the distillation and then reconstitute the volatiles into small aliquots of solvent, with both dichloromethane [41] and diethyl ether [43] used for this purpose. The original SAFE development paper supports both solvent addition protocols; however, they apply to specific sample types. Engel et al. (1999) recommend different sample delivery volumes depending on the sample type being analysed, distinguishing between aqueous samples and oily samples or solvent extracts [71]. As such, pre-extracted samples do not contain the constituents that would remain after extraction, such as proteins, lipids, and sample water. For aqueous samples, such as milk, the recommended workflow is to use direct distillation using SAFE, followed by reconstitution of the thawed volatiles with diethyl ether [71]. When evaluated against the recommended workflow, neither study fully aligned with the standard SAFE procedure or solvent selection. The substitution of diethyl ether for dichloromethane may have several origins, starting with safety concerns. Diethyl ether has very high volatility and extreme flammability, making it a significant concern as a solvent and potentially disallowed in certain laboratories. In comparison, dichloromethane is non-flammable and has low acute inhalation toxicity, making it a lower-risk solvent. Selection of solvents may also be influenced by biases towards certain performance metrics, especially the total number of extracted compounds. In this case, dichloromethane, while still non-polar, is a more polar solvent, allowing it to dissolve a much broader range of compounds. While this may increase total extraction yields, it can lead to co-extraction of lipids and proteins from milk, introducing impurities and increasing matrix complexity for analysis.

##### Concentration of SAFE Extracts

As with any bulk solvent extraction, the volume of organic solvent dilutes the analytes of interest, and a concentration step is necessary before chemical analysis of the extract. The original SAFE methodology employs rotary evaporation for aqueous samples after reconstitution in diethyl ether and Vigreux distillation for extracted model mixtures in solvent [71]. The Vigreux distillation approach was used for concentrating volatiles from UHT milk extracted using SAFE [22], while the extract from sheep’s milk was concentrated using Kuderna-Danish with nitrogen [27]. The Vigreux distillation, although supported by the original method paper, employed a heated water bath and fractional distillation to remove the solvent while minimising loss of volatiles. However, the lighter volatiles, which are representative of the top notes in aroma analysis, would be lost due to the temperatures employed. Additionally, elevated temperatures may promote oxidative and thermal reactions, particularly during the co-extraction of lipids and proteins with dichloromethane. While a concentration step is necessary for accurate analysis, Vigreux distillation may compromise the full aroma profile captured by the SAFE approach. By comparison, the Kuderna-Danish approach mitigates some of the flaws associated with the Vigreux method, as the introduction of a nitrogen stream reduces oxygen levels and lowers the risk of heat-catalysed oxidative reactions. However, the lighter volatiles will still be displaced by the nitrogen stream, still removing the top aroma notes. Both approaches employ fractional distillation as a concentration step; however, the proposed method for concentrating extracts from aqueous samples uses rotary evaporation [71]. Although thermal reactions are minimised, lighter volatiles can still be lost through entrainment with the solvent vapour. When considering SAFE for volatile extraction from milk, it is critical to acknowledge that concentration steps are necessary. However, it will still compromise the product’s overall aroma profile due to loss of volatiles.

##### Extraction Time and Throughput Limitations

The final consideration is the time required to complete the extraction, with the original paper indicating that 4–8 h may be needed [71]. Of the four methods for volatile extraction from sheep’s milk, a preparation time of 12 h per sample is quoted, making SAFE the least efficient method in terms of preparation time. While SAFE may be effective for generating an authentic aroma profile, high-throughput work would not be suited to the extremely long preparation time.

##### Analytical Performance of SAFE

High et al. (2019) found that SAFE exhibited the highest variability among the four methods tested, with an average RSD of 49.7% [27]. In addition to this high average variability, there was a wide range of RSDs across all compounds, from a low of 3.5% for dimethyl sulphide to a high of 150% for octanoic acid [27]. SAFE was well-suited for the extraction of ketones and lactones, with low RSDs typically below 20% for these compounds. The SAFE method extracted 20 compounds with a sensitivity of 627 µg/kg and was more sensitive than the two headspace methods evaluated, likely reflecting its greater recovery of lactones, but it yielded one of the lowest numbers of detected compounds overall. The other three assessed methods were sorptive, indicating that adding sorptive compounds to extraction protocols can yield greater selectivity than traditional bulk solvents. In UHT milk, SAFE was capable of extracting 47 compounds, compared with the SPME approach, which extracted 29 compounds [22]. This paper was not designed to compare methods; therefore, other metrics, such as average RSD and sensitivity, were not presented. In this work, SAFE showed better selectivity for organic acids, alcohols, and aldehydes than SPME; however, it showed lower selectivity towards sulphur compounds. These results show that SAFE, despite some notable drawbacks, remains a powerful method for directly extracting volatiles from milk.

##### Comparison with Sorptive Extraction Techniques

Since SAFE has been compared with sorptive-based methods, it is important to examine them in greater detail. Whilst two sorptive-based methods, HSSE and SPME, have already been discussed in detail, another powerful sorptive method is direct immersion stirbar sorptive extraction. This technique was used only once, compared with SPME, HSSE, and SAFE, for extracting volatiles from spray-dried sheep’s milk [27]. The most comparable methodological approach is HSSE, in which the PDMS-coated stirbar is directly immersed in the liquid sample rather than suspended in the headspace. SBSE benefits from the same factors that give HSSE its higher sensitivity, namely the large surface area-volume ratio between sorptive area and sample. However, by directly immersing the stir bar in the sample, SBSE will increase contact with the heavier semivolatile compounds present in milk, especially lactones and carboxylic acids. The non-polar bias of the PDMS coating resulted in limited selectivity for some lighter volatiles, with dimethyl sulphide undetected by the SBSE approach. Despite this limitation, dimethyl sulphone was detected in substantial concentrations, indicative that the polarity bias does not completely prevent the extraction of polar compounds. When comparing analytical metrics, SBSE showed the highest sensitivity, extracting 657 µg/kg across 45 compounds. The high sensitivity of SBSE was compromised by lactone extraction, as it recovered only 297 µg/kg of non-lactone volatiles, a result comparable to those of the HSSE and SPME methods. When assessing reproducibility, SBSE gave an average RSD of 26.7%, with the lowest RSD of 2.4% for 3,5-octanedien-2-one and the highest being 42.4% for neophytadiene. While SBSE showed greater variability than SPME, it was still substantially more reproducible than SAFE.

While SAFE may have some drawbacks, including lengthy preparation times and poor reproducibility, it remains a powerful technique for aroma analysis when used correctly. Table 3 below indicates the recommended starting conditions for SAFE optimisations, based on the original development paper by Engel et al. (1999) [71] and recent research by High et al. (2019) [27] and Bendall (2001) [73]. When delivering sample aliquots into the SAFE apparatus, the aSAFE modification will improve reproducibility between replicates and across research groups using the technique and is recommended for any SAFE experimentation [72]. Additionally, when conducting SAFE experiments with liquid milk, direct distillation of the product is the preferred approach, as recommended by Engel et al. (1999) [71]. When conducting direct distillation, the best practice is to thaw the volatile distillates, then dissolve them in diethyl ether to complete the distillation. The other two controllable factors, sample volume and temperature, require a complete optimisation trial; however, an initial temperature of 30 °C will limit artefact formation and other thermal interferences during distillation while still allowing efficient extraction. When dealing with sample volume, smaller volumes between 200 and 300 mL will reduce preparation time but may limit SAFE’s comprehensive extraction capabilities. Finally, when concentrating the distillate for analysis, a low-temperature method is needed to prevent artefact formation and analyte loss. As such, rotary evaporation tuned for the removal of diethyl ether is recommended, as this will limit loss and artefacts within the distillate.

#### 4.2.2. Steam Distillation Extraction

The final technique applied for non-target volatile extraction is steam distillation extraction (SDE). This modified steam distillation method involves adding a solvent to extract organic compounds from aqueous mixtures. This involves boiling water to generate steam, which is then blown into a vessel containing the sample and an appropriate solvent. The steam evaporates the solvent, creating a vapour that carries extracted volatile compounds into a condenser. The condensed mixture is collected in a separating funnel to remove any water carried over. Typically, this approach is used to extract organic pollutants from aqueous mixtures rather than for direct volatile analysis [74]. However, the context in which this approach was recently used in dairy applications reflects its origin: it was applied to extract and analyse diterpenoids for authenticity tracing [56]. Although it is used to target terpenoid classes, this analysis shows that SDE can extract a wide range of volatiles from milk, including several flavour-relevant classes of compounds. While analytical measurements such as reproducibility were not assessed, the range of volatiles extracted indicates that SDE can be an effective method for flavour analysis in dairy. As a liquid extraction method, SDE was capable of extracting a range of lactones that were not detected by headspace extractions. The work by Ueda et al. (2016) identified five extracted lactones, showing their suitability for semi-volatilisation analysis [56]. In addition, it was capable of extracting compounds that have a typically strong impact on milk aroma. A range of ketones, aldehydes, alcohols and acids was extracted, including common aroma-active compounds such as hexanal (grassy), 2-tridecanone (waxy) and heptanoic acid (cheesy). As such, SDE offers a suitable balance for extracting both common aroma compounds relevant to flavour analysis and those often overlooked by conventional headspace extraction. The suitability of SDE for dairy analysis may also be linked to the solvent selected in this work, namely, diethyl ether. As previously discussed, diethyl ether is an effective solvent for aqueous samples and is therefore commonly used in SAFE analysis [71]. While SDE is effective for volatile extraction in dairy, there are some limitations worth considering when conducting flavour analysis. As with SAFE, the role of heat must be considered when working with dairy, as prolonged exposure to high temperatures can facilitate thermal degradation reactions in milk. In contrast with SAFE, which uses lower temperatures due to the addition of a vacuum, SDE exposes the milk to high temperatures by blowing steam into the sample. The thermal load on the milk is increased during SDE, making longer extraction times more likely to misrepresent the volatile profile due to artefact formation. Another key limitation is that SDE is exposed to air, introducing oxygen into the sample vessel. In combination with the heat of the steam, oxidative reactions will occur during extraction, potentially resulting in overemphasised cooked and burnt off-odours in the product. Finally, as with all solvent extractions, the concentration of the extract presents significant challenges. In this case, concentration was achieved by partial evaporation, with no indication of the specific protocol used [56]. The heat applied is used to remove the ether; while still gentle, it will promote the loss of extremely light volatiles, limiting SDE’s capacity to extract top aroma notes in dairy.

Overall, the extraction of volatiles from milk reflects a balance among analytical objectives, sample preparation choices, and methodological trade-offs, depending on whether targeted or non-target profiling is required. For the analysis of aroma-active compounds in milk, a combination of headspace and liquid extraction will yield the most complete volatile profile, as headspace methods cannot extract heavier semivolatiles, such as lactones, whilst liquid methods are biased toward the smaller volatile species. A combination of SPME using a DVB/CAR/PDMS coating will extract the broadest range of volatiles and can be readily automated to reduce human error. For liquid extraction, whilst SAFE is a powerful and effective technique, the long preparation time and poor reproducibility make SBSE the more effective approach. As such, future work seeking to conduct a comprehensive volatile analysis of milk should employ SBSE and SPME to generate a comprehensive volatile profile. In addition, studies should conduct full DOE optimisations to examine cross-factor relationships and maximise volatile extraction using the available instrumentation.

## 5. Volatile Profiling Techniques

While sample preparation is the beginning of the analytical stream, an appropriate detection method is necessary to accurately and effectively identify the compounds that influence milk flavour. Due to the complexity of food matrices, a separatory technique is required to isolate volatiles for accurate measurement. To facilitate this, gas chromatography (GC) is used as the “gold standard” for separating volatile compounds before detection. The use of temperature control and specialised capillary column chemistries makes GC a highly adaptable and therefore convenient method for the analysis of a range of volatile compounds. The ability to hyphenate GC with various detection methods, specialised for identification and quantification, has led to a wide range of approaches for analysing volatiles. As GC and the various detectors that can be hyphenated alongside it have been discussed in multiple reviews, this review will summarise trends in column selection and practical principles that will benefit flavour analysis in milk, while leaving detailed detector comparisons to other works.

The first point of variation that is critical when working with GC, and any capillary chromatography, is the selection of the stationary phase used to coat the column interior. The trend observed in this review is that, while specialised columns are used, the majority of milk analyses use one of two standard column coatings: the polar polyethylene glycol (PEG) column, often called a wax column, and the non-polar (5%-phenyl)-methylpolysiloxane or equivalent column, often denoted by a “-5” in the column name. These two column coatings exhibit opposing selectivities, thereby biasing retention toward different functional groups relevant to flavour analysis. The polar PEG column is suitable for separating short-chain fatty acids, alcohols, and aldehydes, thereby reducing co-elution of critical aroma-active compounds. The PEG column, therefore, has better resolving capacity for the aroma compounds present in milk. The 5%-phenyl columns, on the other hand, exhibit a stronger retention bias towards long-chain aldehydes and ketones, aromatic compounds, lactones, and hydrocarbons. These compounds are often oxidation and Maillard products arising from processing, representing the critical flavour compounds to be analysed in studies of off-aromas [4]. Of the papers included in this review, 13 used the PEG phase while 6 used the 5%-phenyl phase, indicating a bias towards the fresh aromas present in milk. The remaining papers used different column chemistries, with one paper each using 100% dimethylpolysiloxane (denoted by a “-1”) [54] and nitroterephthalic acid-modified PEG (an FFAP column) [48]. The different column phases and physical dimensions are summarised in Table 4 below. The dimethylpolysiloxane column is a non-polar column, with a polarity index below that of the 5%-phenyl columns. This column has an even greater retention bias toward long-chain hydrocarbons, so co-elution of aroma-active polar compounds is more likely, limiting the ability to perform detailed volatile profiling with this column. The FFAP column, on the other hand, is less polar than the PEG column but has higher acidity. This enables the column to be used to analyse fatty acids, carboxylic acids, and aromatic acids that may be present in milk. These may be critical flavour compounds, representing both natural and process-induced aromas; however, the column will have reduced resolving capacity for other non-acidic compounds. As such, selection of column chemistries depends on the aroma region of interest: for work on natural flavours in matrices such as raw milks, a PEG column is preferred, whilst for work on process-induced changes and off-aroma analysis, a different column chemistry may be preferred. A final consideration in column selection is the column’s physical parameters. Differences in length, internal diameter, and film thickness can influence separation capacity, peak shape, and analysis time [75]. These factors influence the chromatographic performance but do not fundamentally alter the selectivity of the stationary phase and are therefore not discussed in detail in this review.

Selecting an appropriate stationary phase enables the separation of the individual compounds that impact milk flavour. Hyphenating with a detection method (e.g., mass spectrometry) enables the identification and quantification of compounds; however, relying solely on spectral matching and detector performance can result in poor-quality analysis when analysing volatile compounds. When conducting any GC analysis, standard practice is to include a linear retention index (LRI) method, which uses predictable separation responses and mathematical modelling to provide an additional metric that improves compound identification [76]. Even between technical replicates, minute variations in column flow, sample injection time, temperature, and extraction variations result in slight differences in exact retention times. While some software platforms allow retention time locking to accommodate such drift, using an LRI provides a mathematical method to determine a retention metric independent of minute instrumental variations. Originally developed using an isothermal chromatographic method, the retention index method relies on the consistent separation of *n*-alkanes to assign numeric identifiers to compounds eluting between two alkanes [77]. When an LRI value is correctly determined, multiple laboratories using the same chromatographic conditions can obtain the same value, enabling verification of results across research groups. Isothermal methods are no longer required because the system has been modified to use linear temperature-programmed methods, which still yield reliable separations [78]. However, when working with LRIs, the specific column chemistry employed affects the method’s effectiveness. As LRIs are typically determined using *n*-alkanes, methods using PEG columns may be unable to determine index values for early eluting compounds, due to shorter chain *n*-alkanes co-eluting with the solvent peak (typically *n*-hexane) on the PEG columns. Instead, the recent development of an LRI estimation algorithm based on functional group chemistries can be used to estimate values for compounds separated on polar columns [79]. Algorithm-based LRI estimation has been incorporated into metabolomics workflows, including the establishment of spectral databases [80]. The incorporation of multi-omics approaches can provide standard practices for flavour analysis, improving the reliability of results and increasing the efficiency of multi-institute verification and collaboration of flavour research. The inclusion of LRI values in flavour research aids accurate identification of aroma-active volatiles relevant to flavour analysis. Alongside accurate identification, studies that attempt to quantify these volatiles should follow a standardised approach to ensure accurate quantification. Quantification procedures are often performed using a semi-quantitative approach with an internal standard; however, this approach is inaccurate because it assumes that detectors and compounds interact uniformly [81]. The alternative of constructing calibration curves from authentic standards is expensive and time-consuming, further complicated by the fact that some compounds cannot be purchased as authentic standards. However, authentic standards have been used to determine relative response factors, mathematical determinants that adjust for unique detector-analyte interactions to account for instrumental variation in detection. Trends in the responses of flavour compounds to flame ionisation detectors (FIDs) have been identified, enabling the development of an algorithm to estimate response factors for volatile flavour compounds [82]. This predicted response factor has been incorporated into a standard method, recommended by the International Organization of the Flavor Industry (IOFI) for the identification and quantification of flavour-relevant volatiles [82]. The approach uses the standard method for determining abundance by normalising against the peak area of an internal standard, with the response factor applied multiplicatively to adjust the response. By combining these standard approaches, flavour analysis in dairy will benefit from more accurate identification and quantification of aroma-active volatiles, enabling a deeper understanding of the chemical drivers of flavour.

While gas chromatography is regarded as the “gold-standard” analytical platform for flavour analysis, the complexity of food matrices can hinder the resolution of conventional separation methods [83]. Although traditional GC technologies struggle to separate the complex volatile profile of food effectively, multidimensional GC systems are well established for flavour profiling, with initial reports dating back to the 1970s [84]. Multidimensional gas chromatography primarily employs two techniques: heart-cutting multidimensional gas chromatography (H/C MDGC) for targeted analysis of co-eluting compounds, and comprehensive two-dimensional gas chromatography (GC × GC) for untargeted volatile profiling [85]. Both methods involve an initial separation on a primary analytical column (often 30 m in length), followed by a secondary separation using a column with different, and ideally orthogonal, selectivity. The separation is commonly achieved by first resolving compounds on a non-polar primary column largely according to volatility, followed by further separation on a more polar secondary column that enhances discrimination based on functional-group chemistry. MDGC enhances separation capacity by resolving co-eluting volatiles and providing additional separation space in the secondary dimension.

For targeted analysis, H/C MDGC is preferred because it isolates key region(s) of interest in the first dimension (^1^D), which is then further separated in a long (typically 30 m) second dimension (^2^D) [86]. A microfluidic device, commonly known as the Dean’s switch (DS) in honour of its inventor and marketed under trade names such as Capillary Flow Technology (CFT, Agilent Technologies) and SilFlow (Trajan Scientific and Medical), is used to connect the ^1^D and ^2^D columns [87]. The device contains narrow internal pathways that minimise peak dispersion, while flow switching between the two columns is controlled by an electronic pressure controller (EPC). This arrangement allows narrow effluent bands (typically spanning a few minutes) to be diverted from the ^1^D column and further separated on a full-length analytical column, thereby substantially increasing peak capacity. The trade-offs, however, are longer overall analysis times and a more complex experimental setup: heart-cut windows must be determined in advance, the GC oven typically requires cooling between the ^1^D and ^2^D separations before being ramped again, and a cryotrap is often needed to refocus the transferred band and prevent peak broadening. Although H/C MDGC has been widely applied in flavour analysis, its use in milk analysis remains limited, with most studies focusing on fatty acid methyl esters (FAMEs) and contaminants such as polychlorinated biphenyls (PCBs) [88,89,90,91].

Unlike H/C MDGC, where only selected segment(s) of the ^1^D separation are further separated in the ^2^D column, GC × GC is a technique in which all segments are transferred to the ^2^D column, requiring a rapid means for transferring effluent from the ^1^D column to the ^2^D column [92]. This is achieved by placing a modulator between the two columns, which accumulates and focuses the ^1^D effluent before it is reinjected into the ^2^D column. Although several modulator types have been developed, such as thermal, valve-based, and flow modulators [90], thermal modulation is widely regarded as the most effective modulator for GC × GC [93] and is therefore the most common modulation method used in food analysis [94]. Cryogenic thermal modulators, commercially available under various trade names such as the LECO Dual Jet and the Zoex loop modulator, use liquid N_2_ or CO_2_ jets to trap and re-focus narrow bands of ^1^D effluent before pulsing them into the ^2^D column. This produces the sharp, high-amplitude peaks that enhance GC × GC sensitivity, thereby improving library matching and compound identification. However, the consumable cost and operational complexity associated with cryogen supply have driven the development of consumable-free alternatives, including solid-state thermal modulators (e.g., resistively heated trap designs [95,96], commercialised as the SepSolve INSIGHT modulator) and flow modulators such as forward-fill/flush [97] (LECO FLUX modulator), reverse-fill/flush [98] (LECO Paradigm Shift modulator), and multi-loop splitter-based devices [99]. Flow modulators are attractive for routine food laboratories given their lower running costs and simpler operation, as they require no cryogen logistics; however, they typically yield broader ^2^D peaks and reduced sensitivity for trace odour-active compounds. This is a relevant trade-off in milk aroma analysis, where some quality-impact volatiles (e.g., methional and (*E*,*E*)-2,4-decadienal) could occur at trace concentrations [100].

In addition to the choice of modulation strategy, modulation period (typically 3–8 s) and ^2^D column dimensions must therefore be jointly optimised so that the ^2^D separation completes within each modulation cycle without wraparound. Equally critical is the selection of the stationary phase, since the choice of column pair governs the selectivity of each dimension and how effectively the ^2^D separation space is occupied. In a fully orthogonal system, the two columns separate analytes by independent retention mechanisms, distributing peaks across the ^2^D plane and minimising co-elution. In practice, however, true orthogonality is rarely achieved because the retention of an analyte on any given phase reflects a combination of interactions, and the contributions of volatility and polarity to retention are partially correlated across real analyte sets. Moreover, the term ‘polarity’ itself is an umbrella for several distinct intermolecular interactions, including cavity formation and dispersion interactions (*l*), lone-pair and π-electron interactions (*e*), dipolarity/polarisability (*s*), and hydrogen-bond acidity (*a*) and basicity (*b*), each of which contributes differently to retention depending on the functional groups of the analyte and the chemistry of the stationary phase. The linear solvation energy relationship (LSER) framework, developed by Abraham and co-workers [101,102], decomposes these contributions into corresponding solute (referring to analytes; *L*, *E*, *S*, *A*, and *B*) and solvent (referring to the stationary phase as the partitioning medium; *l*, *e*, *s*, *a*, and *b*) descriptors and has been widely applied to characterise GC stationary phases and to guide the selection of orthogonal column pairs for GC × GC [103].

**Figure 2:**
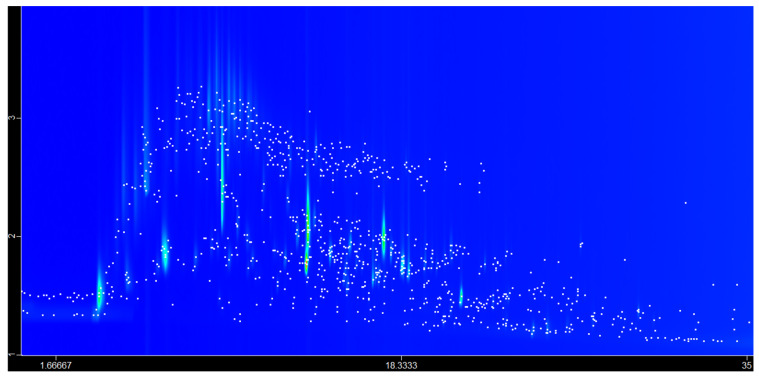
Contour plot of milk analysis using SPME–GC × GC–ToFMS. Each dot represents a peak, with coloured bands representing abundance. Data from unpublished original research.

GC × GC offers significantly higher peak capacity than single-dimensional separations, making it ideal for analysing complex volatile systems, such as those found in flavour analysis [104]. However, the increased complexity of the technique results in equally complex data, with hundreds of peaks in a GC × GC contour plot, presented in Figure 2, often organised by chemical class.

While MDGC is a powerful technique for analysing volatiles, its application in milk analysis is limited. Only one paper has explored the use of MDGC, specifically GC × GC, for analysing milk volatiles [20]. Yue et al. (2015) reported the use of GC × GC coupled with time-of-flight mass spectrometry (ToFMS) to develop a non-target multidimensional approach for determining volatile compounds in dairy products [20]. This research initially employed a conventional GC–MS approach using a low-bleed (5%-phenyl)-methylpolysiloxane nonpolar column. The GC × GC was carried out using the same ^1^D column with a (50%-phenyl)-methylpolysiloxane mid-polar column as the ^2^D column. This arrangement is beneficial for resolving the chemical-class clusters relevant to milk flavour, such as separating alcohols, aldehydes, and ketones from one another, and resolving aromatic from aliphatic hydrocarbons. The reversed-polarity configuration (polar ^1^D × non-polar ^2^D) is less commonly applied in dairy analysis, but it can be advantageous for separating closely related polar oxygenates that are difficult to resolve on non-polar ^1^D columns, such as short-chain alcohols and aldehydes. Selectivity tuning through alternative ^2^D chemistries (e.g., ionic liquid phases for free fatty acids [105] or trifluoropropyl-substituted phases for compounds containing electron-rich functional groups [106]) represents a further avenue for targeted enhancement of milk aroma profiling, although such configurations remain underexplored in the dairy literature.

Volatile identification in this study combined NIST library spectral matching with linear retention indices (LRI) generated from an *n*-alkane mixture, providing an additional metric for confirming compound identities [20]. Peak matching was carried out using LECO ChromaTOF software version 4.4, and compounds with a library match score ≥ 80% were considered tentatively identified. Manual inspection was also used to further ensure the quality of the VOC identifications. Using conventional GC–MS, 52 compounds were identified, of which acids (11) and aromatic hydrocarbons (11) were the most predominant chemical classes identified in the milk. However, when GC × GC is used, 207 volatiles were identified, 107 of which had not been reported in milk before, with aliphatic hydrocarbons (69), aromatic hydrocarbons (42), and ketones (28) being the three most predominant classes. GC × GC resolved more compounds across all chemical classes than the conventional one-dimensional separation, including 8 ethers that were previously undetected in the GC–MS approach. Together, these findings demonstrate a compelling case for the use of multidimensional separations in dairy analysis, enabling deeper investigation of critical aroma compounds and other volatiles for quality control.

For volatile profiling, separation-based methods are used because they are well-established in the literature. The ability to separate and isolate volatiles for identification and quantification can provide high confidence in profiling. However, separation requires sample preparation and long analysis times, creating a barrier for analytical process monitoring. Direct injection methods and non-destructive profiling approaches can be used to rapidly identify volatiles in various samples. One such approach, which has been applied to milk, is proton transfer reaction time-of-flight mass spectrometry (PTR–ToFMS). This method uses direct injection of headspace samples into a drift tube, where H_3_O+ ions collide with VOCs. This is a soft ionisation approach that generates protonated molecular ions ([M + H]+) with minimal additional fragmentation. While this method can be used for rapid analysis and simple spectral identification workflows, it remains limited in scope. As there is no separation step, VOCs with the same mass are indistinguishable unless high-resolution ToFMS is used. Additionally, simple spectra can be beneficial for rapid analysis and identification; however, the lack of unique fragment patterns under hard ionisation limits compound identification compared with approaches that utilise LRIs and calibration standards for further confirmation. Finally, PTR–ToFMS can be used for semi-quantification; however, proton affinity and reaction kinetics can prevent full protonation and, therefore, the quantification of VOCs. In dairy and flavour analysis, PTR–ToFMS is primarily used for fingerprinting, process monitoring, and classification rather than comprehensive volatile profiling. However, it can be used to supplement the profiling of conventional separation methods, given the high resolution of time-of-flight mass spectrometers. Corvino et al. (2025) recently used PTR–ToFMS and GC–MS to generate profiles of bovine milk [50]. The PTR-ToFMS method generated 188 mass peaks, including both VOCs and VOC fragments, using a 25 s infusion from the headspace into the flight tube. In comparison, the GC–MS approach consisted of a 45 min SPME extraction followed by a 42 min separation protocol, yielding 13 identified compounds. Although the PTR–ToFMS method provides a faster approach and more abundant mass peaks, confirming compound identities can be difficult without LRI or fragmentation patterns. Importantly, the authors acknowledged that PTR-ToFMS is an excellent complementary tool to conventional separation technologies, highlighting the benefits of rapid profiling when combined with GC–MS identification capabilities.

## 6. Sensory Analysis and Instrumental Correlation

While volatile profiling is highly useful for assessing chemical changes, it does not provide an accurate perspective on the flavour effects of these volatile compounds. As such, the analysis of milk products requires appropriate sensory methodologies to link volatile compounds directly to flavour effects. While full human assessments provide the highest-quality data for sensory analysis, not all laboratory environments are suitable for panel training and for setting up sensory evaluation booths. In such cases, electronic sensor arrays, such as e-noses and e-tongues, have seen increased use for their ability to perform classification and screening of flavour in complex matrices. While sensor arrays cannot replace human perception, they may provide some diagnostic information that can be correlated with more comprehensive volatile profiling to model the impact of volatiles on overall flavour. Table 5 highlights sensor array and human assessment methods that have recently been used to perform flavour analysis across a variety of milk matrices.

As highlighted in Table 5, the inclusion of human sensory analysis alongside volatile analysis is limited. Additional sensor data to support volatile changes are usually collected using a complementary electronic sensor array, thereby limiting a true understanding of the impact of volatiles on sensory outcomes. Among the three studies that employed human sensory methods, two used quantitative descriptive analysis (QDA) [8,54], whilst one used gas chromatography coupled with olfactometry (GC–O) [48]. QDA utilises a trained human panel to assess relevant sensory properties such as aroma, flavour and overall acceptance in one study [54] and a more in-depth analysis comprising aftertaste (milk flavour, sweetness, fat-sense), butter, texture, sourness, off-flavour, aroma, milk flavour and saltiness [8]. QDA is a powerful sensory technique that enables differentiation of subtle changes in key sensory properties and requires intensive training to ensure that the panel assessing the product can adequately identify them [107]. While trained human panellists are selected for their sensitivity to specific sensory changes, maintaining these panels can be time-consuming and expensive, creating significant barriers for some research groups. Electronic sensors can be used to identify chemical differences within samples, after which human sensory analysis can be used to determine the flavour impacts associated with the chemical differences. Sensors may detect differences between samples on a chemical basis; however, these do not directly relate to flavour changes. As such, human sensory methods are always needed in flavour analysis workflows. It has been shown that e-Nose and e-Tongue can identify differences between samples with a capacity comparable to that of a trained QDA panel [8]. While useful, electronic arrays cannot capture key sensory changes; human panels are essential for interpreting which sensory properties change and how these changes affect overall acceptance. The effectiveness of electronic sensor arrays is evidenced by the number of studies that incorporate these techniques into analytical workflows, with equal numbers employing exclusively electronic and exclusively human analysis. In the use of electronic sensor technologies, the selection of appropriate sensors will determine the method’s effectiveness in terms of both sensor type and targeted compounds to be detected. In this review, two eNose arrays and two eTongue arrays are discussed, with electrodes selected to respond to a range of analytes. The first eTongue method used metal-oxide-semiconductor (MOS) sensors to conduct voltametric analysis of compound classes, with specific electrodes exhibiting reactivity potentials toward key flavour classes. This specific sensor array used a series of electrodes (Pt, Au, Pd, W, Ti and Ag) to generate electrochemical fingerprints dependent on proteins, peptides, salts, organic acids and oxidation products, which allow for the sensor to discriminate between samples processed with different UV-C and pressure conditions [5]. These sensors provide an instrumental approach for identifying differences in compounds that influence taste and texture, indicating the need for human assessment to discern the corresponding perceptual changes. In contrast, the second eTongue method utilised chemically modified field-effect transistors (CHEMFETs) in a potentiometric approach for fingerprinting. The CHEMFET approach responds more directly to ionic and acid-base changes in the milk than to redox interactions in the sample. The CHEMFETs sensor array uses specialised sensors that detect voltage changes indicative of pH modifications. These membrane interactions indicate protein denaturation, ionic strength, and, via interactions with small molecules, oxidation products. Both the eTongue and the trained QDA panel discriminated among the processed samples. They independently identified the treatment that most closely matched the taste profile of the control milk, which was formulated to approximate raw milk while remaining safe for consumption [8]. However, the match was based on a statistical interpretation of chemical changes, without insight into the specific sensory drivers behind them. The QDA panel identified four factors of importance when evaluating dairy products: the desirable factors of “milk flavour”, “butter”, and “aroma” and the undesirable “off-flavour”. When analysing the different processing conditions, the same temperature and time conditions used in the eTongue analysis were found to have the highest sensory scores for the desirable factors whilst showing the lowest for the undesirable factors [8]. This indicates that while the eTongue can reflect sensory outcomes, it cannot be used to identify the perceptual drivers and sensory outcomes underlying these differences. Both eTongue approaches demonstrate that sensor arrays are effective for chemically based discrimination in sensory analysis; however, because this assessment is decoupled from human perception, the technique cannot be used to infer consumer preferences in product development. The inclusion of e-tongue techniques should be considered only a routine instrumental quality control approach, not a replacement for sensory evaluation or analytical flavour research. At this stage, e-tongue systems provide a rapid screening tool for monitoring product consistency, detecting batch-to-batch variation, and identifying potential deviations from expected quality profiles. They may be useful for complementing detailed physicochemical tests and non-volatile analyses of milk, particularly in quality assurance settings where rapid, repeatable measurements are required. However, any assessment of sensory perception must be conducted using human panels, including trained panels for descriptive analysis and consumer studies to determine sensory acceptance. The rapid screening capacity of e-tongue systems may support quality assurance when linked with comprehensive sensory testing, but their outputs should be interpreted as instrumental indicators rather than direct measures of human flavour perception. Therefore, e-tongue methods should not be used in isolation to conduct flavour research or to make conclusions about sensory quality without validation against human sensory data. In addition to eTongue workflows for taste and texture analysis, an understanding of volatile aroma compounds and their influence on flavour is also of great importance and can be analysed using eNose arrays.

The two eNose arrays employed different sensor configurations but shared the same analytical method. The MOS sensors are heated in air to promote oxygen adsorption and rapid redox reactions and are then exposed to the milk headspace [108]. The volatile compounds then undergo redox reactions at the sensor surface, which are calibrated to detect different redox species generated by varying compound classes of interest [109]. Redox reactions affect the resistance measured by the sensor, which is how eNose systems quantify the relative abundance of compound classes [110]. In the two eNose applications for milk analysis presented in this review, sensors were used to detect sulphur-containing organic compounds, methane, hydrogen, alcohols, and various hydrocarbon species; one method used 10 sensors [7] and the other used 14 sensors [5]. The larger array featured sensors designed to analyse fossil-fuel VOCs, alcohols, ketones, aldehydes, combustible gases, and organic solvent species. This array encompasses a much broader range of volatile species that may be relevant, likely resulting in a more complex VOC profile in milk. In both cases, the eNose system’s performance was comparable to that of conventional GC–MS analysis. When determining whether a food additive affected the volatile profile of milk, both eNose and volatilomic analysis indicated no significant differences in the volatile composition [7]. When comparing processing effects on milk aroma, both eTongue and eNose identified the same sample as significantly different, indicating the suitability of both methods for rapid “flavour” analysis as complementary techniques to separation-based profiling [5]. In this case, GC–MS data were not analysed using multivariate statistics, so a direct comparison between a conventional approach and a MOS sensor approach was not possible. However, as with eTongue, the sensor-based approaches cannot provide insight into sensory outcomes associated with volatile changes. It is important to note that, as currently available, electronic sensors can only be used for pattern recognition and classification. They cannot replace human sensory analysis, as they lack the capacity to detect and interpret the subtle changes and interactions that inform the perception of aroma and flavour.

Human sensory perception is highly sensitive to aroma, with olfactometry analyses revealing identified odours in the absence of complementary VOCs [111]. Certain odour compounds can be present at very low concentrations, below the limit of detection for mass spectrometry. However, they may still provide a significant aromatic character, requiring human olfactory perception to detect their presence in milk. This indicates that even sensitive instrumental methods cannot comprehensively capture the complexity of aroma as detected by human panellists. Additionally, incorporating human sensory techniques enables descriptive analysis of aroma compounds, leading to a better understanding of the impact of volatiles on product acceptance. An effective method for incorporating human panellists alongside separation techniques is GC–O, which allows human “sniffers” to detect aroma compounds as they are separated, thereby enabling the identification of important aroma compounds within the total aroma profile [112,113].

GC–O combines the separation capacity of gas chromatography with the sensitivity of the human olfactory system, through the use of a trained sniffing panel, to identify individual aroma characters within complex food matrices. Typically, these systems are combined with an additional detector, either flame ionisation detection (FID) or mass spectrometry (MS), to enable qualitative compound identification and determination of aroma character [114]. In GC–FID/O systems, qualitative analysis is initially performed using an independent GC–MS system, with retention time and LRI matching used to assign aroma descriptors to the separated compounds. GC–O can be conducted using a range of experimental designs, with aroma extract dilution analysis (AEDA) and modified frequency (MF) being the two most common approaches [113]. These two methods employ different approaches to achieve the same end goals, thereby determining the relative importance of each aroma compound within the overall aroma profile. AEDA is the most commonly used method in GC–O workflows, employing physical dilution to reduce the concentration of individual aroma compounds progressively. AEDA uses a panel of two trained sniffers to identify aromas as they are separated, indicating when each aroma is present. AEDA is initially conducted on undiluted samples to identify all possible aroma compounds. The base sample is then diluted, either manually using solvents or by adjusting the splitting ratio, several times to bring compounds below their detection threshold. [115]. The theory of AEDA states that the most important compounds are those that retain an aroma character at progressively higher dilutions [116]. However, this does not reflect the importance of low-concentration compounds with large aroma contributions that are mechanically diluted away, thereby preventing detection. The level of dilution at which an aroma persists is referred to as the flavour dilution (FD) factor, which provides a numerical comparison for determining relative importance. AEDA workflows utilising HS-SPME should be handled with care, however, as previous work has shown that direct adjustment of the GC injector split ratio yields the highest linearity between FD and peak area [117]. However, this directly contradicts the mechanics of HS-SPME, which should always be conducted in splitless injection mode [118]. Dilutions should be prepared by serial dilution with an appropriate solvent. This may lead to issues with matrix effects, further complicating the use of HS-SPME in AEDA workflows. Additionally, AEDA depends on the sensory detection threshold of the individual compounds and may not be linearly correlated with analyte concentration [119,120]. This leads to the erroneous assumption that physical concentration is the underlying mechanism by which aromas change. AEDA is also constrained by the high sample-preparation burden, necessitating an intensive workload to prepare the required range of samples [121]. Additionally, the panel of sniffers for AEDA needs to be well-trained and highly sensitive to the sample’s aromas. This leads to smaller panel sizes of two or three sniffers, meaning individual sensitivity variances will have a larger overall impact on data quality. MF offers a common alternative to AEDA that eliminates some of its drawbacks.

MF is a detection frequency method that relies on large panels of 6–12 sniffers, each of whom describes the aroma and rates its intensity as it elutes [122]. The inclusion of a large number of panellists allows MF to reduce data variance caused by individual panellist sensitivity [123,124]. In contrast to AEDA, no dilutions need to be prepared, with each sniffer being exposed to the unaltered sample. In traditional frequency-based detection methods, aroma importance is determined by the number of panellists who can identify each aroma [125]. These methods rely on the erroneous assumption that detection frequency is a direct indicator of the aroma’s potency [126]. MF still considers the number of detections, but also includes an additional factor based on the average intensity of each aroma as determined by the panel [127]. Overall, while AEDA is a well-established technique for aroma analysis, the limitations of a small number of sniffers, olfactory fatigue, and long analysis sessions with multiple dilutions likely necessitate multi-day experiments. AEDA has many potential sources of error. As such, aroma analysis should be conducted using MF techniques to improve statistical significance and reduce experimental load, thereby limiting between-day variation in samples. The equation determines the relationship between intensity and detection frequency:

(1) $$MF\left(\%\right)=\sqrt{\frac{n}{N}+\frac{I_{Avg}}{I_{Max}}}\times 100\%$$

where *n* and *N* represent the number of detections and the number of panellists, and *I_Avg_* and *I_Max_* represent the average and maximum intensity of the aroma. Ranking aromas by calculated MF value allows the determination of the most important compounds in the aroma profiles [128,129,130]. When determining the importance of aroma, traditional frequency-based detection methods are biased by the number of detections and do not account for the intensity of individual aromas. This will lead to compounds that may have been detected by most of the panel being rated as most important, despite only just being detected, preventing the ranking of aroma compounds with equal detection numbers [131].

In a study by Pandohee et al. (2022), the GC–O methodology used was AEDA, conducted on coffee-flavoured UHT milk [48]. This paper followed good practice, using manual dilution of the product with deionised water and splitless injection for SPME desorption. Additionally, the method used an initial GC–MS analysis to determine compound identity and to develop an LRI. GC–O was then conducted, using LRI values to match aroma descriptors to mass spectral matches. While some good practices are used, the inherent flaws of AEDA methodologies become evident, particularly in GC–O data collection. The authors indicate that they used two panellists, as is standard in AEDA. The samples tested were the undiluted product and serial dilutions up to a dilution factor of 1024, yielding 11 total samples per panellist, with each panellist repeated at four different time points. This is further compounded by the fact that each analysis is completed in triplicate, resulting in 132 individual sniffing sessions per panellist. Assuming that multiple sessions were not conducted consecutively, between-day variations in analysis will have impacted the data quality. If sessions had been consecutive, sensitivity fatigue would have reduced the panellists’ capacity to identify subtle aroma changes [132]. Despite methodological limitations, this work indicates the importance of GC–O in flavour analysis, as it enabled the identification of key aroma compounds that influence the overall profile of a flavoured milk product. This shows both the effectiveness of GC–O as a method for qualitative and quantitative aroma analysis and its capacity to handle complex food matrices. However, this study highlights the lack of integration between sensory and instrumental analysis. While a link between compound identities and aromatic character is attempted, the statistical analysis relies exclusively on the aroma data and their changes. No attempt is made to quantify the volatiles and associate their continuous change during storage with the changing aroma character. By linking quantifiable changes in volatiles to observed aroma effects, it may be possible to identify multifactor interactions that shape aroma rather than merely assuming that single compounds are responsible for complex aroma character. This modification would enable the identification of mechanisms driving aroma change, creating actionable insights for manufacturers.

The use of single-dimensional separation for olfactometry analysis is well established across a range of food products, with some work conducted in dairy matrices. However, as with volatile profiling, the complexity of food samples can reduce the effectiveness of these methods. While deconvolution algorithms may reduce the impact of co-elution, in aroma profiling, the more dominant aroma will overpower the senses, preventing the detection of some compounds. As such, multidimensional separation has been used in olfactometry, with comprehensive aroma profiling conducted using GC × GC–O [133]. As with volatile analysis, the incorporation of a modulation device and orthogonal column chemistries allows separation of co-eluted aroma compounds, improving detection of low-intensity aromas that more intense ones mask. However, this technique demands significant effort from panellists due to the high elution speed. Heart-cutting multidimensional gas chromatography (H/C MDGC) provides a viable alternative by isolating complex aroma regions and using the 2D column for further separation [134,135]. This method reduces the panellist’s workload while offering a more comprehensive aroma profile than single-dimension techniques. While not applied to milk, multidimensional gas chromatography coupled with olfactometry provides an extremely sensitive approach for separating and identifying aroma compounds that influence flavour. In combination with methods such as MF, these techniques may lead to more complex aroma profiles, providing greater insight into how volatiles affect flavour. However, the use of advanced data collection methods is only useful when combined with appropriate statistical analysis.

## 7. Data Analysis and Chemometrics

While analysing flavour requires selecting appropriate analytical and sensory methods, understanding flavour requires appropriate data analysis techniques. The use of multivariate statistics enables researchers to draw more complex conclusions by assessing correlations between chemical and sensory changes. To ensure high-quality data, a consistent analytical approach needs to be established. The complex chemical data required for flavour analysis suggest that generic statistical approaches may be insufficient. In contrast, a well-designed chemometric approach can integrate these data streams to yield meaningful conclusions. Chemometrics is a multidisciplinary field that employs advanced statistical methods to analyse chemical data, addressing both descriptive and predictive problems, making it suitable for fingerprinting and predicting chemical changes [136,137,138,139,140]. In milk flavour analysis, chemometrics can establish baseline volatile profiles (otherwise known as an initial fingerprint) and predict how chemical changes reflect quality loss. Due to the complexity of chemical data, multivariate techniques such as principal component analysis (PCA), hierarchical clustering analysis (HCA), and partial least squares (PLS) are commonly applied. Unsupervised methods like PCA and HCA are used initially, with PCA identifying key features for classification and HCA detecting qualitative groupings via dendrograms [141,142,143]. The combination of PCA and HCA provides initial screening information on how sample classes may be grouped and which variables contribute to that variation. Supervised methods, such as partial least-squares discriminant analysis (PLS-DA) and orthogonal PLS-DA (OPLS-DA), have been applied to build classification and prediction models using the training data [141,144]. As computational methods evolve, machine learning is increasingly integrated into chemometric analysis. Beginning with descriptive statistics and general significance tests, such as Analysis of Variance (ANOVA), provides an initial indication of chemical changes, which can then be analysed using PCA and HCA to identify drivers of change between sample types. Here, sensory data could be effectively incorporated, particularly using PCA, which enables researchers to identify samples with higher consumer-preference scores and the potential chemical drivers of those preferences. In the studies presented in this review, chemical and sensory data are typically analysed separately using distinct PCA plots, limiting the ability to conclude the chemical drivers of sensory perception. The standard approach in the included studies is to construct PCA plots from sensory data, whether from electronic or human sensors, to indicate perceptual differences between sample groups. The sensory changes are then discussed in relation to GC–MS data, correlating certain classes with potential aroma impacts [8]. While this approach links sensory outcomes to chemical data, separating the two data streams precludes direct identification of which chemical changes drive sensory differences. Another possible approach is to use regression analysis to show how certain chemical changes over time and correlate with changes in sensory perception. This has been demonstrated using microbial cell counts in milk, showing that increased bacterial species directly affect consumer sensory ratings [54]. While conducting individual regression analyses for 15 to 20 volatiles would not be overly difficult, studies employing more sensitive methods or multidimensional separations may be required to handle 45 to 219 volatiles, each of which would require a regression analysis [20,27]. It is far more efficient to use large-scale modelling approaches, such as PCA, to conclude how volatile compounds directly affect flavour perception.

**Figure 3:**
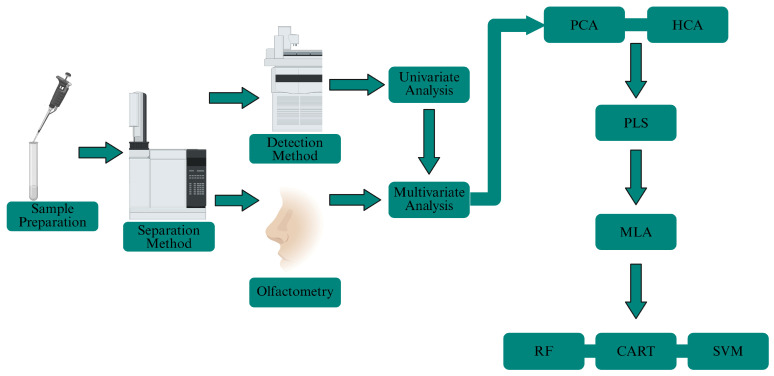
Analytical workflow for milk aroma analysis. Created in BioRender. FRC, C. (2026) https://BioRender.com/7bs3bzk, accessed on 11 May 2026.

When considering the use of models in statistical analysis, machine learning algorithms (MLAs) provide a powerful means of combining multiple data streams into a unified model. Like PCA and PLS-DA, MLs can be supervised or unsupervised, enabling many ways to implement them in a data analysis workflow. Of the various MLAs available, flavour analysis would benefit from pattern recognition algorithms, as they are best equipped to identify key chemical markers that influence sensory outcomes. Three pattern recognition algorithms: support vector machine (SVM), classification and regression tree (CART), and random forest (RF), have gained prominence in applying machine learning to food systems. These algorithms function as classification models, similar to PLS-DA, for the qualitative identification of discrete groups. These methods have been applied in studies for identifying geographical origin, detecting adulteration, quality control, and predicting food properties across a wide range of products, including fruits [145], herbs [146], meats [147], liquors [148], beverages [149], and dairy [150]. Although these algorithms are commonly used in omics-based analyses, their application to complex food analysis is growing. These advanced methods facilitate the analysis of complex chemical data and the assessment of how changes in volatility may affect milk flavour. By training MLAs on a range of sample types and by combining volatile changes with sensory outcomes, researchers and manufacturers could analyse a subset of samples and model consumer outcomes. Whilst not a stand-in for full consumer-driven product development, the inclusion of MLAs and model-based statistics will help support initial formulation efforts and quality monitoring programmes. The three key MLAs, SVM, CART, and RF, have different applications and drawbacks when correlating volatile analysis with sensory outcomes. SVM can be used for both regression and classification, using volatile data to predict sensory outcomes or to classify samples according to sensory scores. However, SVMs do not provide information about which volatiles drive the sensory outcomes, limiting actionable product insights. In contrast, CART can also be used for regression and classification analyses but provides a clearer link between volatile data and sensory scores. CART is also capable of revealing threshold effects, determining which sensory outcomes are influenced by specific volatile concentrations. CART can be limited by high overfitting, especially with large volatile datasets and small sensory datasets. CART can also overlook multi-compound interactions, instead preferring single-factor predictors of sensory outcomes. RF is more robust to overfitting than CART and provides relative importance rankings, which can be very important for identifying driver compounds behind sensory outcomes. Variable importance may be biased to more abundant compounds, not necessarily those that carry the greatest sensory impact. Additionally, RF cannot account for thresholds and synergistic interactions among volatiles when determining sensory outcomes. The proposed data analysis workflow for chemometrics-based flavour analysis is summarised in Figure 3 below. By following this workflow, the correlation between sensory and volatile data will improve. This will allow further improvements in understanding how various factors and changes affect the delicate flavour of milk.

## 8. Future Perspectives

While a substantial body of work has examined aroma compounds in milk, several methodological and conceptual gaps continue to limit progress toward a mechanistic and comparable understanding of flavour development. Addressing these gaps will require more standardised, systematically designed, and integrative research approaches.

A key priority is the development of standardised methodologies for volatile extraction and analysis, particularly for solid-phase microextraction (SPME). Current studies often optimise individual parameters in isolation, despite well-established interactions among extraction time, temperature, sample volume, and salt concentration. Future work should adopt multivariate experimental designs (e.g., response surface methodology or factorial designs) to simultaneously optimise these parameters and report them in a reproducible framework. Crucially, optimisation should be validated across a range of milk matrices, including skim, reduced-fat, and full-fat systems, to ensure method robustness and comparability between studies. In parallel, systematic benchmarking of alternative extraction techniques (e.g., dynamic headspace, stir bar sorptive extraction) under controlled conditions would enable the identification of matrix-appropriate and analytically efficient workflows, rather than relying on method selection by convention.

Advances in chromatographic separation also represent a significant opportunity for improving milk volatile characterisation. Although one-dimensional gas chromatography (1D GC) remains widely used, future studies should more consistently adopt multidimensional gas chromatography (e.g., GC × GC) to improve separation capacity and detect co-eluting compounds that may contribute to aroma. Method development in this area should include explicit comparison of column chemistries, modulation conditions, and detection systems, with performance evaluated using metrics such as peak capacity, compound coverage, and reproducibility. Furthermore, the integration of gas chromatography–olfactometry (GC–O) with multidimensional separations should be expanded, with structured protocols that link odour events to chemically resolved peaks, thereby strengthening the identification of aroma-active compounds rather than total volatile profiles.

A further critical direction is the improved integration of sensory and instrumental datasets. Future studies should move beyond parallel but independent analyses and instead employ unified experimental designs in which volatile profiling and sensory evaluation are directly aligned in terms of sample handling, experimental conditions, and statistical treatment. The application of multivariate data analysis, including partial least squares regression and emerging machine learning approaches, should be used not only for pattern recognition but for explicitly modelling relationships between chemical composition and sensory perception. Importantly, these approaches should be implemented with appropriate validation strategies (e.g., cross-validation, external validation datasets) to ensure that derived relationships are robust and generalisable.

The increasing use of machine learning and other advanced data-driven approaches offers additional opportunities to address complex, nonlinear relationships among milk composition, processing conditions, and flavour outcomes. Future research should focus on interpretable models that can identify key predictive compounds or compound classes driving sensory attributes, rather than purely predictive “black box” models. Integration of multi-omics datasets (e.g., lipidomics and proteomics alongside volatilomics) may further enhance mechanistic understanding, particularly in linking precursor availability to aroma formation pathways during processing and storage.

Finally, studies employing electronic sensor arrays should more clearly define their role within aroma analysis frameworks. Rather than being used as standalone tools for flavour characterisation, these systems should be validated against human sensory data and calibrated using chemically resolved datasets. Future work should prioritise hybrid approaches in which electronic sensors are used for rapid screening or quality control, while sensory panels and instrumental analyses provide mechanistic interpretation. Establishing such complementary roles will improve both the interpretability and practical applicability of sensor-based measurements in dairy systems.

Collectively, the adoption of standardised methodologies, multidimensional analytical techniques, and integrated sensory–instrumental frameworks will be essential to advance reproducibility, comparability, and mechanistic understanding in milk aroma research.

## 9. Conclusions

This review highlights that progress in understanding milk aroma is constrained less by the absence of analytical tools and more by how those tools are applied and integrated. A central finding is that methodological choices in sample preparation, volatile extraction, and chromatographic separation strongly influence the reported volatile profiles, limiting comparability across studies and complicating the interpretation of flavour outcomes.

Among available approaches, gas chromatography–based techniques remain fundamental; however, their effectiveness depends heavily on experimental design. Multidimensional chromatographic approaches offer clear advantages for resolving complex milk matrices and should be prioritised when comprehensive volatile characterisation is required. Likewise, no single sample preparation method is universally optimal, with technique suitability dependent on matrix composition and analytical objectives. This underscores the need to select methods based on defined analytical goals rather than convention.

A key contribution of this review is the identification of a persistent disconnect between chemical and sensory analyses in milk flavour research. Instrumental profiling alone is insufficient to explain consumer perception, and studies that do not account for aroma activity risk misrepresent the compounds most relevant to flavour. Integrating sensory methodologies, particularly olfactometry and structured sensory evaluation, with instrumental data is therefore essential for identifying the true drivers of aroma. Electronic sensor arrays may provide value as complementary tools, but their outputs require validation against both human perception and chemically resolved data to ensure meaningful interpretation.

Finally, this review emphasises that advances in data analysis, including multivariate and machine learning approaches, offer significant potential but must be applied within well-integrated experimental frameworks. The ability to link volatile composition to sensory perception depends not only on analytical sensitivity but also on the alignment of chemical, sensory, and statistical methodologies.

Collectively, this work provides a framework for more consistent and mechanistically informative aroma analysis in milk, highlighting the need for standardised methodologies, improved sensory–instrumental integration, and application-driven method selection. By clarifying these priorities, the review helps advance more reproducible, comparable, and consumer-relevant flavour research in dairy systems.

## Figures and Tables

**Table 1:** Summary of techniques for extracting volatiles from milk from the literature (2010–2026).

Extraction Method	Extraction Mode	Extraction Phase	References
Solid-phase microextraction	Headspace	DVB/CAR/PDMS, DVB/PDMS, CAR/PDMS	[3,5,8,20,22,27,48,50,51,52,53,54]
Headspace stirbar sorptive extraction	Headspace	PDMS	[27]
Static headspace	Headspace	Not Applicable	[55]
Dynamic headspace	Headspace	Not Applicable	[7]
Stirbar sorptive extraction	Liquid Immersion	PDMS	[27]
Solvent-assisted flavour evaporation	Solvent	Dichloromethane	[22,27]
Steam distillation extraction	Solvent	Steam Distillation	[56]

**Table 2:** Optimum Conditions for HS-SPME Analysis of Liquid Milk Volatiles.

Condition	Recommended Value
Temperature	35–40 °C
Equilibration Time	30 min
Extraction Time	30 min
Sample Volume	5–10 mL
Salt	Anhydrous NaCl, 1.7–3.4 g
Fibre Coating	PDMS/CAR/DVB

**Table 3:** Optimum Conditions for SAFE of Liquid Milk Volatiles.

Condition	Recommended Value
Sample Delivery	Automated SAFE
Extraction Type	Direct Distillation
Solvent	Diethyl Ether
Sample Volume	100–200 mL
Temperature	30 °C
Concentration Method	Rotary Evaporation, 15–25 °C, 120–140 mmHg

**Table 4:** Summary of chromatographic methods used for analysis of volatiles from milk.

Column Chemistry	Column Dimensions (L (m) × ID (mm) × *d*_f_ (µm))	Reference
PEG	30 × 0.25 × 0.25	[5,27,51,53]
PEG	30 × 0.32 × 0.25	[8]
PEG	30 × 0.32 × 0.5	[50]
PEG	60 × 0.25 × 0.25	[22]
(5%-phenyl)-methylpolysiloxane	30 × 0.25 × 0.25	[3,7,20,52]
(5%-phenyl)-methylpolysiloxane	30 × 0.32 × 1	[56]
(5%-phenyl)-methylpolysiloxane	15 × 0.53	[55]
100% dimethylpolysiloxane	60 × 0.32 × 0.25	[54]
Nitroterephthalic acid modified polyethylene glycol	20 × 0.25 × 0.25	[48]

**Table 5:** Summary of sensory methods used in various milk products.

Milk Product	Assessment Type	Method	Panel/Sensor Type	Reference
Raw Milk	Electronic Sensor	e-Nose	Inert Metal Oxide	[7]
Raw Milk	Electronic Sensor	e-Nose, e-Tongue	Inert Metal Oxide	[5]
Raw Milk (HHP)	Electronic Sensor	e-Nose, e-Tongue	Inert Metal Oxide	[5]
Raw Milk (UV-C)	Electronic Sensor	e-Nose, e-Tongue	Inert Metal Oxide	[5]
Pasteurised Milk	Electronic Sensor/Human Sensory	e-Tongue, QDA	CHEMFETs, Trained	[8]
Pasteurised Milk	Human Sensory	QDA	Trained	[54]
Coffee-Flavoured Milk	Human Sensory	GC–O	Trained	[48]

## Data Availability

No new data were created or analysed in this study. Data sharing is not applicable to this article.

## Abbreviations

The following abbreviations are used in this manuscript:

AEDA	Aroma Extract Dilution Analysis
ANOVA	Analysis of Variance
CAR	Carboxen
CART	Classification and Regression Trees
CHEMFET	Chemically Modified Field-Effect Transistor
DOE	Design of Experiment
DSI–UHT	Direct Steam Injection UHT
DVB	Divinylbenzene
FAME	Fatty Acid Methyl Ester
GC	Gas Chromatography
GC × GC	Comprehensive Two-Dimensional GC
H/C MDGC	Heart-Cutting Multidimensional GC
HCA	Hierarchical Clustering Analysis
HHP	High Hydrostatic Pressure
HSSE	Headspace Sorptive Extraction
HS–SPME	Headspace Solid-Phase Microextraction
HTST	High Temperature Short Time
IND–UHT	Indirect UHT
IOFI	International Organization for the Flavor Industry
MF	Modified Frequency
MLA	Machine Learning Algorithm
MOS	Metal Oxide Sensor
MS	Mass Spectrometer
O	Olfactometry
OPLS–DA	Orthogonal Partial Least Squares Discriminant Analysis
PCA	Principal Component Analysis
PCB	Polychlorinated Biphenyls
PDMS	Polydimethylsiloxane
PEG	Polyethylene Glycol
PLS	Partial Least Squares
PLS–DA	Partial Least Squares Discriminant Analysis
PTR–ToFMS	Proton Transfer Reaction Time of Flight Mass Spectrometry
QDA	Quantitative Descriptive Analysis
RF	Random Forest
RI	Retention Index
RSD	Relative Standard Deviation
SAFE	Solvent-Assisted Flavour Evaporation
SBSE	Stirbar Sorptive Extraction
SDE	Steam Distillation Extraction
SVM	Support Vector Machine
ToF	Time of Flight
UHT	Ultra-High Temperature
UV–C	Ultraviolet Light–C
VOC	Volatile Organic Compound
